# ﻿Generic concepts and species diversity within the Gynoxyoid clade (Senecioneae, Compositae)

**DOI:** 10.3897/phytokeys.234.107750

**Published:** 2023-10-10

**Authors:** Belen Escobari, Thomas Borsch, Norbert Kilian

**Affiliations:** 1 Botanischer Garten und Botanisches Museum Berlin, Freie Universität Berlin, Berlin 14195, Germany; 2 Herbario Nacional de Bolivia, Universidad Mayor de San Andres, Casilla, La Paz, 10077, Bolivia; 3 Institut für Biologie, Systematische Botanik und Pflanzengeographie, Freie Universität Berlin, Berlin 14195, Germany

**Keywords:** Andes, Asteraceae, character evolution, chloroplast capture, EDIT Platform, morphology, phylogeny, taxonomic backbone, taxon concepts

## Abstract

The Gynoxyoid clade of the Senecioneae (Asteraceae) until now included the five genera *Aequatorium*, *Gynoxys*, *Nordenstamia*, *Paracalia* and *Paragynoxys* as diagnosed using selected morphological characters. In their pre-phylogenetic circumscription, the genera *Aequatorium* and *Paragynoxys* were considered to inhabit the northern Andes in contrast to *Nordenstamia* and *Paracalia* that occur in the central Andes. The most species-rich genus, *Gynoxys*, was believed to be distributed throughout the Andes. We use a recently established plastid phylogenomic framework that rendered *Gynoxys* paraphyletic to further evaluate the delimitation of genera in the Gynoxyoid clade. We examine the morphological variation of all members of the Gynoxyoid to identify characters potentially informative at genus level. This results in a matrix of eleven, mostly multistate characters, including those originally used to diagnose these genera. The ancestral character state inference displays a high level of homoplasy, but nevertheless supports the recognition of four genera. *Aequatorium* is characterised by white radiate capitula. *Paracalia* and *Paragynoxys* share white flowers and floral characteristics, such as flower opening and length of disc flowers lobes, as plesiomorphic states, but differ in habit (scandent shrubs vs. trees). *Paracalia* also retained white flowers, but its two species are characterised by the absence of outer phyllaries. The genera *Gynoxys* and *Nordenstamia* comprise species with yellow capitula which appear to be a derived feature in the Gynoxyoids. The genus *Nordenstamia*, with eight species, is synonymised under *Gynoxys* since molecular evidence shows its species nested within various parts of the *Gynoxys* subclade and the morphological variation of *Nordenstamia* falls well within that of *Gynoxys*. With the goal to assign all species to four genera (*Aequatorium*, *Gynoxys*, *Paracalia* and *Paragynoxys*), we assess the states for the eleven characters for all members of the Gynoxyoids and generate new ETS and ITS sequences for 171 specimens belonging to 49 species to further support their generic placement. We provide a taxonomic treatment for the four genera recognised here including amended diagnoses and morphological descriptions. Furthermore, a species-level taxonomic backbone is elaborated for all genera using electronic tools that list 158 currently accepted names and synonyms (209 names in total) with the respective protologue and type information, as well as notes on the current understanding of species limits. Eleven names are newly synonymised, two are lectotypified and eight are newly transferred to other genera.

## ﻿Introduction

The Gynoxyoid group is a New World clade of the subtribe Tussilagininae (Senecioneae, Asteraceae) that was estimated to comprise around 150 species in five genera (Nordenstam et al. 2009). The clade includes shrubs, trees and, more rarely, lianas, growing at the higher elevations of the Andes, in humid mountain forests, subalpine forests and in the paramo. Originally, Jeffrey (1992) suggested the existence of this group of putatively related genera, based on cylindrical anther-collars, polar endothecial thickening and high chromosome numbers, based on x = 10. He included *Capelio* B.Nord. (as *Alciope* DC.) from South Africa (Nordenstam 2002), the Andean genera *Paracalia* Cuatrec., *Paragynoxys* (Cuatrec.) Cuatrec., *Gynoxys* Cass. and *Aequatorium* B.Nord. and the Caribbean genus *Herodotia* Urb. & Eckm. Subsequently, Robinson et al. (1997) restricted the group to the South American genera and pointed out that it is characterised by a chromosome number of 2n = 80. The *Roldana* clade, sister to the *Gynoxys* clade (Pelser et al. 2007, 2010), in contrast, has a chromosome number of 2n = 60 (Jeffrey 1992). These high chromosome numbers can be explained by ancient polyploidisation in the Tussilagiinae. The genus *Nordenstamia* Lundin was later erected to accommodate certain species previously placed in *Aequatorium* and *Gynoxys* (Lundin 2006).

The first phylogenetic data for the Gynoxyoid group were provided by Pelser et al. (2007) in the context of inferring relationships within the Senecioneae, based on sequences of the nrITS region. The authors resolved a clade with the genera *Aequatorium*, *Gynoxys*, *Nordenstamia* and *Paragynoxys* and found *Nordenstamia* (2 species) nested within *Gynoxys* (4 species). Pelser et al. (2010) extended the taxon sampling with a representative of *Paracalia* and increased the number of molecular markers (nrITS and nrETS and plastid *ndhF*, *psbA-trnH*, *rbcL*, 5′ and 3′ *trnK*, *trnL* and *trnL-F* regions) and essentially confirmed their earlier results. Recently, Escobari et al. (2021) provided a comprehensive plastid phylogenomic framework, based on 17 complete plastid genomes representing all five genera and close American relatives within the Tussilagiinae. Their results corroborated the Gynoxyoid group as monophyletic with high support. The three representatives of the genus *Nordenstamia* were found nested within a broadly paraphyletic genus *Gynoxys*. Additionally, the plastid genome sequence of *Paracaliajungioides* appeared as sister to *G.baccaroides* and *G.violacea* within *Gynoxys*, whereas *P.pentamera* was retrieved as sister to all other members of the Gynoxyoids. The second diverging clade was comprised of the monophyletic *Paragynoxys* and the only representative of the genus *Aequatorium*.

The Gynoxyoid group represents one of the speciose Andean plant lineages and, thus, contributes significantly to the high species diversity and endemism in the Andes as one of the global biodiversity hotspots (Myers et al. 2000; Padilla-Gonzalez et al. 2021). The uplift of the Andes led to shifts in ecosystem barriers (Luebert and Weigend 2014; Bacon et al. 2022) and enabled the creation of new habitats (Colwell et al. 2008; Moreira-Munoz et al. 2020; Perez-Escobar et al. 2022) which seem to have triggered rapid speciation of Andean plants (e.g. Madriñán et al. 2013; Zhang et al. 2021; Perez-Escobar et al. 2022). Amongst the studies focusing on the evolution of Andean plant groups (see Hughes and Atchison (2015)), several dealt with genera of the sunflower family, such as *Diplostephium* Kunth (Vargas et al. 2017), *Espeletia* Mutis ex Bonpl. (Pouchon et al. 2018) and *Loricaria* Wedd. (Kandziora et al. 2022). In all three cases, the authors reported low genetic distances, complicating the study of species relationships and species limits. Moreover, frequent events of reticulate evolution and incomplete lineage sorting have been reported from rapidly evolving Andean plant groups (Garcia et al. 2014; Vargas et al. 2017; Schley et al. 2021; Kandziora et al. 2022). Low genetic distances were also observed amongst plastid genomes in the Gynoxyoid clade in our previous study (Escobari et al. 2021). Consequently, we demonstrated that complete plastid genome sequences, including the more variable intron and spacer partitions, were needed to achieve resolution at species and even genus level. The results of Escobari et al. (2021) underscored that *Gynoxys* is not monophyletic as currently circumscribed and that an evaluation of morphological characters hitherto used to diagnose the genera of the Gynoxyoid clade in an evolutionary context is warranted.

Cassini (1827) described the genus *Gynoxys* as having a tree-like habit, opposite leaves, the presence of an indumentum on the lower leaf surface, corymbiform capitula and the apex of style branches vested by papillose hairs as diagnostic characters. Weddell (1855) subdivided *Gynoxys* into two sections: one with radiate and the other with discoid capitula, which has lately been adopted by Correa (2003). The first taxonomic treatment including a larger number of species was made by Herrera (1980) who dealt with the 30 species distributed in Peru. That author redefined the genus by having usually opposite leaves, an indumentum on the lower leaf face, discoid or radiate capitula with up to 32 yellow disc flowers, an inconspicuously sagittate anther base and a conical, hispid and caudate style-branch apex. According to published regional checklists, *Gynoxys* is distributed from Bolivia to Venezuela at altitudes between 1600 and 4700 m above sea level and estimated to comprise about 180 species (Brako and Zarucchi 1993; Jorgensen and Leon-Yanez 1999; Beck and Ibañez 2014; Bernal et al. 2019).

*Paragynoxys* was first described by Cuatrecasas (1951) as Seneciosect.Paragynoxys, but raised to generic rank shortly thereafter (Cuatrecasas 1955). It is characterised by a tree- or shrub-like habit, subcoriaceous petiolate alternate leaves, a corymbose-paniculate terminal synflorescence, few-flowered discoid capitula, white corollae with the limb divided to its base, conical style-branches and a distribution in Colombia and Venezuela. The only taxonomic revision by Correa (2003) recognised 12 species and extended its diagnosis by having radiate capitula with five or more inner phyllaries and up to 12 flowers.

*Paracalia* was segregated by Cuatrecasas (1960) from *Paragynoxys* because of its scandent habit, smaller leaves and involucre lacking outer phyllaries. The genus comprises two species distributed in Bolivia and Peru (Cuatrecasas 1960; Nordenstam 2007; Hind 2007).

*Aequatorium* was published by Nordenstam (1978) to accommodate two shrubby species with alternate leaves, a rusty tomentum of stellate hairs, white corollae, sagittate or auriculate anther bases and blunt style-branches apices. Subsequently, several new species were added (i.e. Díaz-Piedrahita and Cuatrecasas 1990; Jeffrey 1992; Díaz-Piedrahita and Cuatrecasas 1994; Nordenstam 1997), resulting in an ongoing discussion on morphological features suitable for circumscribing the genus (see Nordenstam 1997). Based on the presence of stellate hairs and the differently-shaped involucre, Jeffrey (1992) transferred GynoxyssectionPraegynoxys to *Aequatorium*. Nordenstam (1997) concurred with this hypothesis and divided *Aequatorium* in two subgenera. Aequatoriumsubg.Aequatorium included species with (generally) alternate leaves, peltate trichomes forming two layers, white flowers, apically obtuse style branches; distributed in Ecuador and Colombia. Aequatoriumsubg.Praegynoxys included species with opposite or alternate leaves, irregular branching trichomes, absence of the overlying brownish tomentum, yellow flowers and apically pointed style branches and distributed in Argentina, Bolivia, Peru and southern Ecuador. Interestingly, he even suspected that the latter subgenus may be closer to *Gynoxys* than to *Aequatorium*. These concerns were taken up by Lundin (2006), who raised Aequatoriumsubg.Praegynoxys to a genus of its own, *Nordenstamia*, including 14 species.

Since the establishment of *Gynoxys*, the first genus in the clade, almost 200 years ago, new species continue to be described in this conspicuous Andean plant group (Cuatrecasas 1950, 1951, 1954, 1955; Robinson and Cuatrecasas 1992; Beltrán and Baldeón 2009; Beltrán and Calvo 2020). However, monographic work aiming at a synthesis of taxonomic data was largely limited to *Gynoxys* and *Paragynoxys* (Herrera 1980; Robinson and Cuatrecasas 1984; Nordenstam 1997; Correa 2003) or to geographically-confined areas (Dillon et al. 1993; Nordenstam and Lundin 1999; Badillo et al. 2008; Beck and Ibáñez 2014; Avila et al. 2016). The considerable species number, the shallow morphological differentiation within the clade and the absence of a robust phylogenetic hypothesis added considerable uncertainty and instability to the circumscription of the genera of the Gynoxyoids, which has found its expression in frequent transfers of species between genera. A consistent taxonomic synthesis is, therefore, needed for the whole Gynoxyoid clade.

The availability of electronic sources for names and protologue citations (IPNI, www.ipni.org; TROPICOS, www.tropicos.org), as well as online access to digitised type specimens (JSTOR Global Plants, https://plants.jstor.org/) and electronic tools to support the taxonomic workflow (EDIT Platform; Berendsohn (2010)) has facilitated the way taxonomic treatments are undertaken. More recently, a comprehensive name source is available through the World Flora Online Plant list which is regularly updated (worldfloraonline.org). Therefore, names can be imported into an electronic taxonomic working tool so that the actual taxonomic research can focus on checking validity of names and testing taxon concepts at species level. At the same time, the taxonomic workflows are revolutionised by structured data (Kilian et al. 2015) and evolutionary approaches to investigate species limits (Stuessy and Lack 2011; Marhold et al. 2013).

For the Gynoxyoid clade, we have taken on the task to check all names and to present a consistent classification at species level as a baseline hypothesis for the whole clade using the available data. While our approach is still largely based on morpho-species, it utilises some phylogenetic data that could be generated for specimens representing part of the species. Our goal was to elaborate an expert-revised taxonomic backbone for a plant group throughout its range of distribution in the sense of the workflow of the World Flora Online (WFO; see Borsch et al. (2020)), ideally including all validly-published names assigned to a status as accepted name or synonym. Such a taxonomic backbone also provides the best possible taxonomic knowledge in time as this is needed for conservation status assessments, biodiversity monitoring etc.

Considering this situation, the aims of this investigation are: [1] to revise the generic classification of the Gynoxyoids making use of molecular (plastome and nrDNA) and morphological data and [2] to provide a revised species inventory of the Gynoxyoids for the entire range of distribution.

## ﻿Materials and methods

### ﻿Plant material and sources for specimen data

The study was based on plants observed, collected and photo-documented in the field during three collecting trips in Bolivia and Peru, as well as physical specimens loaned to B from AAU, F, G, K, LPB, MA, MO, NY and P (Thiers, continuously updated). Specimens that were physically examined are listed in Suppl. material 1. In addition, high resolution digital images of herbarium specimens, in particular types, were consulted online either accessed through JSTOR Global Plants (https://plants.jstor.org/), GBIF (https://www.gbif.org/) or directly through online databases of the individual herbaria.

### ﻿Sources of names and compilation into a checklist of the species of the Gynoxys clade

The species inventory of the Gynoxyoids was built in a database using the EDIT Platform for Cybertaxonomy (Berendsohn 2010), based on imports of names and associated data (authors, protologue citations) from the International Plant Names Index (IPNI) (https://www.ipni.org/) supplemented by TROPICOS (https://tropicos.org/home), the Global Asteraceae Database (https://www.Asteraceae.org/aphia.php?p=stats) and the World Flora online (http://www.worldfloraonline.org/)

### ﻿Definition and assessment of morphological characters and states

The first round of assessing the morphological variation in the Gynoxyoid group included all species of the genera *Aequatorium*, *Nordenstamia*, *Paracalia* and *Paragynoxys* and a representative selection in terms of morphological diversity of *Gynoxys* species, altogether 65 species. We examined the diagnostic characters stated in the protologues and in other studies of the five genera, but also compared specimens to detect morphological variance to develop a list of characters and their states. For this investigation, a character state was considered taxonomically relevant and selected for further processing if its expression marked morphological discontinuities at supra-specific level. For each such character, unordered categorical states were defined following the terminology by Roque et al. (2009) and Beentje (2010). In cases where a more detailed homology statement was needed due to conflicting or unclear use of character definitions or terms, a description and illustration were included. For later reconstruction of character evolution, a specimen-based matrix of characters and states suitable for reliable delimitation and characterisation of supra-specific entities was constructed using the specimens included in the plastid phylogenomic analysis of Escobari et al. (2021). For certain characters, for example, the plant habit, the respective states were recorded from literature if not given on the specimen label.

### ﻿Ancestral character state reconstruction

Only Bayesian trees obtained from complete plastome sequences with indels coded and alignments manually corrected as provided by Escobari et al. (2021) were used as the hypothesis of the phylogenetic relationships in the Gynoxyoids, because lack of resolution rendered the use of nrDNA marker trees impossible. The reconstruction of character states at ancestral nodes was performed with a Bayesian approach using BayesTraits version 2.0 (Pagel and Meade 2006), which uses a selection of post-burn-in trees obtained from the t.files of the Bayesian analysis. This random selection of 800 of the total of 1600 post-burn-in trees taken from Escobari et al. (2021) was obtained through Mesquite version 3.7 (Maddison and Maddison 2021). The file stating the relevant nodes of the tree to be addressed by the analyses of BayesTraits was generated with TreeGraph v.2.14beta (Stöver and Müller 2010). The inference of the ancestral character state reconstruction was performed using the reverse jump MCMC approach with 5,050,000 iterations, with a burn-in of 50000, a sample frequency of 1000 and, following the recommendation by Pagel and Meade (2006), a hyper-prior where the mean of the exponential is drawn from a uniform 0–100 distribution. TreeGraph v.2.14beta (Stöver and Müller 2010) was used to plot the results from the BayesTraits output log file with the function Import BayesTraits data on the Bayesian major consensus tree. We excluded the other genera of the Tussilagineae that were present in the plastid phylogenomic investigation, considering that the outgroup sampling in their dataset is incomplete with respect to the morphological diversity.

### ﻿Extraction, amplification and phylogenetic tree inference of nuclear ribosomal DNA

To achieve a better overview on species-level phylogenetic relationships within the Gynoxyoid clade and to test if groups of samples identified with the same species name appeared in terminal subclades, 171 samples belonging to 50 species (Suppl. material 1) were included into an extended molecular dataset. These samples were selected to cover morphological and geographical variation as much as possible and also included the samples that were already part of the plastid phylogenomic study. The nrITS and nrETS regions were used as they provided some variable and informative characters in a short marker that was possible to sequence with little effort per sample. Additionally, by representing the nuclear genome, the dataset could be used to test for incongruence between trees inferred from different genomic compartments. Plastid regions often applied to assess the tree space of speciose clades (Mansion et al. 2012) were not suitable in the Gynoxyoid clade due to extremely low genetic distances (Escobari et al. 2020). Genomic DNA was extracted using the CTAB method by Doyle and Doyle (1987), with three fractions for each sample as modified by Borsch et al. (2003). PCR amplification of ITS followed White et al. (1990), ETS was amplified with the primers AST-1 (f) and 18-S-ETS (r) (Markos and Baldwin 2001), following Pelser et al. (2010). PCR was performed in a peqSTAR Thermocycler 1107D (PeqLab, Erlangen, Germany). The PCR products were electrophoresed on 1.5% agarose, the bands were cut out and cleaned with the GenepHlow Gel/PCR kit (Geneaid, New Taipei, Taiwan). Samples were sequenced by Macrogen Europe (Amsterdam, The Netherlands). Sequence files were aligned using MAFFT v.7.394 (Katoh and Standley 2013) and manually edited using PhyDE version 0.9971 (Müller et al. 2010), following the rules of Löhne and Borsch (2005). Indels were coded as binary characters using the simple-indel-coding method (Simmons and Ochoterena 2000) in SeqState version 1.4.1 (Müller 2005). Altogether, 146 ETS and 166 ITS sequences were newly generated and the sequences were deposited in the European Nucleotide Archive (ENA) using the annonex2embl submission pipeline (Grünstäudl 2020) and can be retrieved from ENA under study number PRJEB53579 (https://www.ebi.ac.uk/ena/submit/webin/study/PRJEB53579).

Phylogenetic trees were inferred from the ITS, ETS and a concatenated matrix of both belonging to the corresponding samples in the plastid tree presented in Escobari et al. (2021). A Bayesian analysis was performed with MrBayes v.3.2.6 (Ronquist and Huelsenbeck 2003), using four parallel Markov Chain Monte Carlo (MCMC) runs for a total of 50 million generations under the GTR+G+I model. The convergence of the Markov chains was checked with Tracer v.1.7 (Rambaut et al. 2018). The initial 25% of all trees were discarded as burn‐in and the remaining trees were used to summarise the 50% majority consensus tree.

### ﻿Assignment of all species to genera and evaluation of taxon concept at species level

Despite the extended nuclear ribosomal sequence dataset generated in this investigation, not all species could be included into phylogenetic analysis. This was largely due to the unavailability of suitable material, for example, in species only known from type or historical specimens. We, therefore, used our set of eleven morphological characters with their states in conjunction with the results from ancestral state reconstruction, to assign all species to a genus and, in the case of *Gynoxys* species, also to informal infrageneric groups of morphologically similar species that can be used as a hypothesis on close relationships. The genera and informal infrageneric entities were described and a taxonomic key for their determination was created. At species level, all protologues were consulted to check for the correct typification of names. Type specimens of all names, with the exception of only a few unavailable ones (indicated in the taxonomic treatment part, below), were examined from high resolution digital images provided by JSTOR Global Plants, GBIF and the herbarium websites of individual herbaria. The digital images of type specimens were referenced in the checklist to the type citation. Where necessary, new combinations were made and names were lectotypified. As a general principle, a morpho-species concept, delimiting species purely based on morphological discontinuities, was applied. Type specimens and additional specimens (see Suppl. material 1) were examined to assess the qualitative differences and possible infraspecific variation with the aim to hypothesise a name as accepted or as a synonym. The citation of authors follows the international standards by Brummitt and Powell (1992); the citation of publications follows BPH (Bridson et al. 2004) and TL-2 (Stafleu and Cowan 1976–1986; Stafleu and Mennega 1992–2009); the latter was also consulted for actual publication dates. Accepted names were provided with full synonymies and type citations. Type specimens that were online include only the herbarium acronym. Specimens that were physically examined are marked with (!).

## ﻿Results

### ﻿Morphological characters of taxonomic relevance on supra-specific level

**Figure 1.**
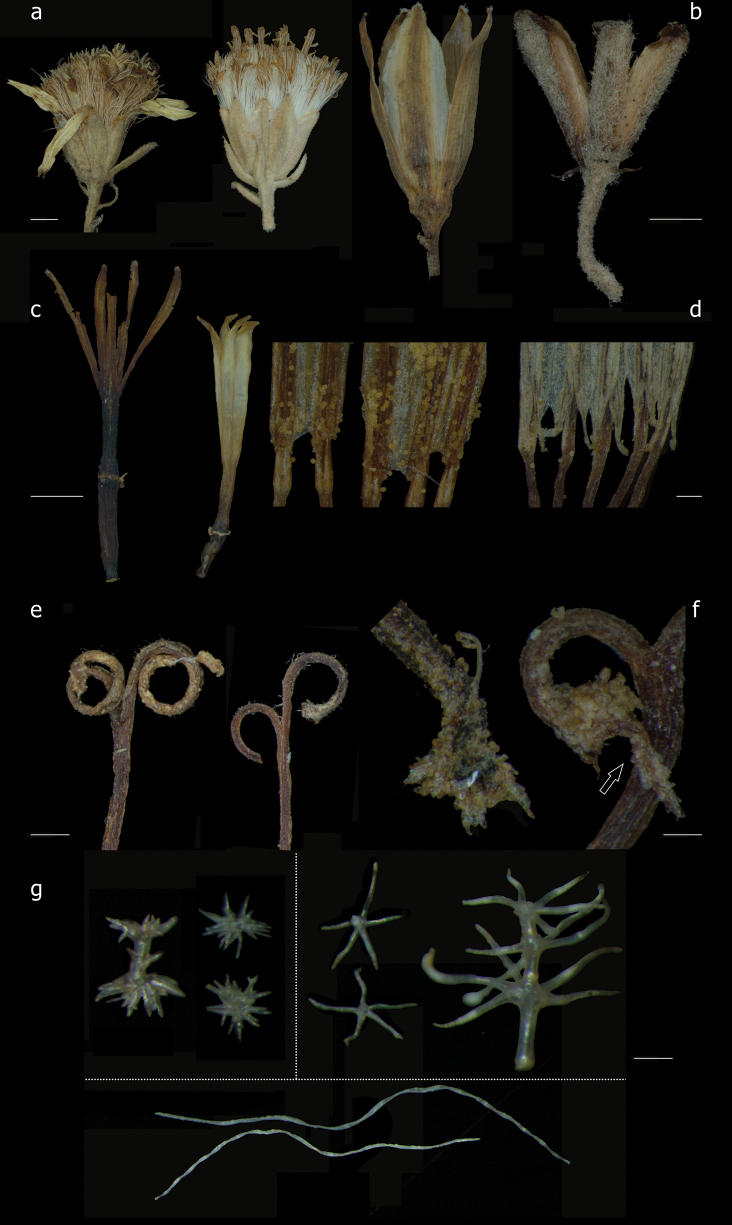
Characters and states from floral morphology and indumentum for the Gynoxyoid clade **a** capitula: radiate (*Gynoxyscalyculisolvens* left), discoid (*G.longifolia* right) **b** involucral outer phyllaries: absent (*Paracaliapentamera* left), present (*Paragynoxysmartingrantii* right) **c** ratio corolla lobe/tube length: deeply lobed (*Paragynoxysvenezuelae* left) (ratio > 0.6), shortly lobed (*G.asterotricha* right) (ratio < 0.6) **d** anther-base shape: obtuse (*Paracaliajungioides* left), sagittate (*G.ignaciana* right) **e** lenght of style branches: large (*P.jungioides* left), short (*G.ignaciana* right) **f** style branches apex shape: rounded (*G.ignaciana* left), acute (*G.baccharoides* right) **g** trichome architecture: multicellular hairs (*A.jamesonii* left above, *N.kingii*, right above), unicellular hairs (*G.violacea* below line). Scale bars: 2 mm (**a–c**); 200 μm (**d**); 500 μm (**e**); 100 μm (**f, g**).

The evaluation of morphological characters with respect to discontinuities at supra-specific level resulted in a matrix of eleven characters. These characters and their states are defined in Table 1 and, where appropriate, illustrated in Fig. 1.

**Table 1.** Morphological characters selected for the ancestral character reconstruction analysis with their respective character abbreviation (Abbr.) and character states with a respective abbreviation and definition when needed.

Character	Abbr.	Character states
Plant habit	H	tree (T), shrub (S), scandent (C)
Phyllotaxis	P	alternate (A), opposite (O)
Trichome architecture	T	Trichomes absent (G), unicellular hairs (S), multicellular hairs (M)
		Unicellular hairs: unicellular simple hair.
		Multicellular hairs: branched or unbranched hairs. Differences between multicellular hairs were avoided since several types of these can be present in a same specimen (Fig. 1F).
Corolla colour	CF	white (W), yellow (Y)
		This character state describes both ray and disc flowers since it is always shared by both flower types in a capitulum.
Outer phyllaries	OP	absent (A), present (P)
		As outer phyllaries were considered all phyllaries attached at the base of the involucrum and not at the peduncle of the capitulum
Number of inner phyllaries	InP	≤ 5 (F), 6–8 (M)
		The following categories are based on the stability of a defined number of phyllaries for the genera
Radiate flowers	RF	absent (D), present (R)
		The states implicitly define the architecture of the capitulum. The absence of ray flowers (0 = A) represents a discoid capitulum (Fig. 1A). A number > 0 represents a radiate capitulum
Number of disc flowers	DF	≤ 8 (F), > 8 (M)
		The following categories are based on the stability of a defined number for the genera
Ratio corolla lobe/tube length	Rat	≤ 0.6 (S), > 0.6 (D)
		This character describes the opening depth of the corolla. Length of lobes in relation to the length of the corolla tube (shortly vs. deeply lobed corolla) (Fig. 1C).
Anther-base shape	AB	sagittate (S), obtuse (O)
		The base of the anthers is defined as obtuse when no appendage can be distinguished (Fig. 1e). We ignored the difference between acute (small appendages) vs. sagittate (large appendages) since both can be present in a same specimen and this may be unstable depending on the state of the specimen
Style branch apex shape	SA	acute (A), rounded (R)
		The style branch apex is described as acute when the branches tips have a conspicuously pointed tip (Fig. 1D). We use rounded in a wider sense also including an apex described as truncate, as the presence of papillose hairs makes the distinction unreliable

### ﻿Morphological characterisation of the members of the Gynoxys clade

Our evaluation for consistent presence and absence of sets of diagnostic character states in Gynoxyoid species resulted in the recognition of four morphologically and phylogenetically defined genera. The morphological matrix with the diagnostic characters applied to the genera and species of the Gynoxyoids represented in the sampling for the plastome tree is given in Table 2.

**Table 2.** Assessment of characters and their states for the species within the Gynoxyoid clade as included in the phylogeny inferred by Escobari et al. (2021). The codes of characters and states are noted in Table 1, (?) indicates missing data.

Species	H	P	T	CF	OP	InP	RF	DF	Rat	AB	SA
*Gynoxysmegacephala*	S	O	M	Y	P	M	D	M	S	S	A
*Nordenstamiacajamarcensis*	T	O	M	Y	P	M	R	F	S	O	R
*Gynoxysignaciana*	S	O	S	?	P	M	R	M	S	S	R
*Gynoxyslongifolia*	S	O	S	Y	P	M	D	M	S	S	R
*Nordenstamiarepanda*	T	A	M	Y	P	M	R	F	S	S	A
*Nordenstamiakingii*	S	A	M	Y	P	M	R	M	S	S	A
*Gynoxysviolacea*	S	O	S	Y	P	M	R	M	?	S	R
*Gynoxysasterotricha*	S	O	S	Y	P	M	R	M	S	S	A
*Gynoxysbaccharoides*	S	O	S	Y	P	M	R	M	S	S	A
*Gynoxyscalyculisolvens*	S	O	S	Y	P	M	R	M	S	S	A
*Gynoxystomentosissima*	S	O	S	Y	P	M	R	M	S	S	A
*Gynoxysmandonii*	T	O	S	Y	P	M	R	M	S	S	A
*Aequatoriumjamesonii*	S	O	M	W	P	M	R	F	S	O	R
*Paragynoxysmartingrantii*	T	A	M	W	P	F	D	F	D	O	R
*Paragynoxysvenezuelae*	T	A	M	W	P	F	D	F	D	O	R
*Paracaliajungioides*	C	A	S	W	A	F	D	F	D	O	R
*Paracaliapentamera*	C	A	G	W	A	F	D	F	D	O	A

The first of these four genera is *Aequatorium* with all species sharing the combination of multicellular trichomes, radiate capitula, white flowers, a low number of disc flowers (< 8) and an obtuse shape of the anther base. Diagnostic for this genus is the unique combination of white flowers and radiate capitula.

Further genera are *Paracalia* and *Paragynoxys*, the species of which are differentiated from the other Gynoxyoid genera by a deep-lobed corolla, white flowers and discoid capitula. *Paracalia* can be distinguished from *Paragynoxys* by a scandent habit, absence of outer phyllaries and a central Andean distribution. In contrast, *Paragynoxys* has a woody habit, an involucrum with outer phyllaries and a north-Andean distribution.

The genus *Nordenstamia* cannot be delimited morphologically. The presence of stellate hairs by which this genus was originally distinguished from *Gynoxys* (Lundin 2006) is not only highly variable amongst the *Nordenstamia* species, but also shared with many *Gynoxys* species. If *Nordenstamia* is included in *Gynoxys*, this genus can be differentiated from all the others by the combination of yellow flowers and a shallowly divided disc corolla.

*Gynoxys* is notably the most diverse taxon within the Gynoxyoid clade, displaying a wide range of morphological variation. Within the genus, three informal groups can be discerned, based on distinct characteristics, including phyllotaxis, the number of ray flowers and the type of trichomes. The first group encompasses species with discoid capitula. In contrast, the second group comprises species with multiseriate stellate hairs, primarily featuring alternate leaves. Finally, the third and largest group is characterised by opposite leaves, radiate capitula and simple hairs.

### ﻿Phylogenetic trees inferred from nuclear ribosomal markers

In addition to the trees of the Gynoxyoids, based on a representative set of complete plastid genome sequences (Escobari et al. 2021), this study attempted to provide further phylogenetic evidence from nrDNA, amongst many others also including the same set of samples present in the plastid tree. Three phylogenetic analyses were performed, based on the ribosomal nuclear markers ETS, ITS and a concatenation of both (Suppl. material 2). In contrast to the tree, based on the plastid genome (Suppl. material 2: appendix 2a), the Bayesian ETS and ITS trees are poorly resolved (Suppl. material 2: appendix 2d). In all trees, the members of the *Gynoxys* clade form a single polytomy. The sister group relationship between the two species of *Paragynoxys* is the only clear congruence between the two nuclear ribosomal trees (however, with low support in the ITS inference) and is, moreover, in conformity with the plastid genome tree. Only the ETS tree resolved the two species of *Paracalia* as a (moderately supported) clade (Suppl. material 2: appendix 2c), whereas they were resolved in separate clades in the ITS (Suppl. material 2: appendix 2b) and in the concatenated ETS+ITS tree (Suppl. material 2: appendix 2d).

### ﻿Character evolution in the Gynoxyoids based on the phylogenetic hypothesis of the plastome tree

**Figure 2.**
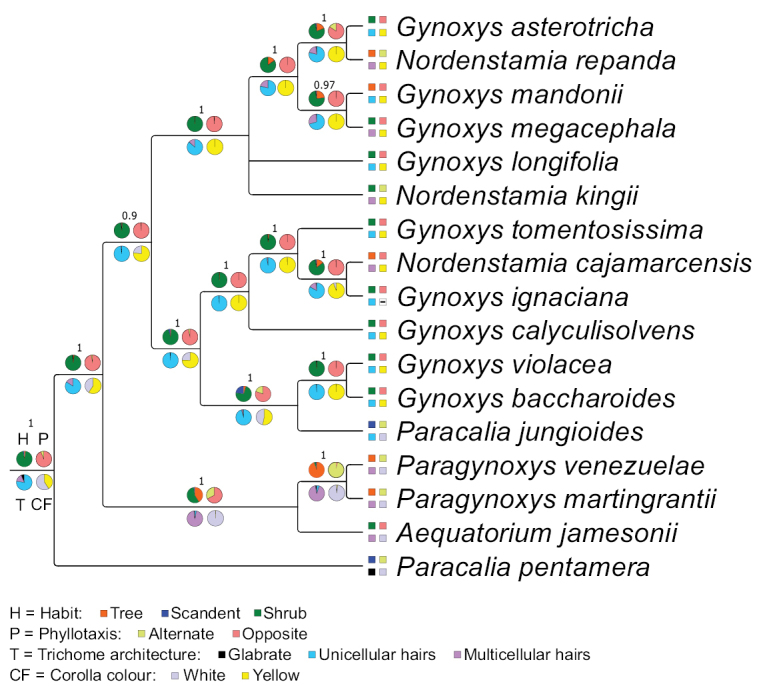
Bayesian inference of ancestral character state reconstruction of four morphological characters of the Gynoxyoid clade in the consensus plastome tree by Escobari et al. (2021). Each pie chart represents a single character and each colour represents a character state as described in the legend. The actual state of the characters is represented by boxes next to the species names. The pie charts at the stem of the tree show the character abbreviations as mentioned in Table 1. Missing data are represented as (-).

**Figure 3.**
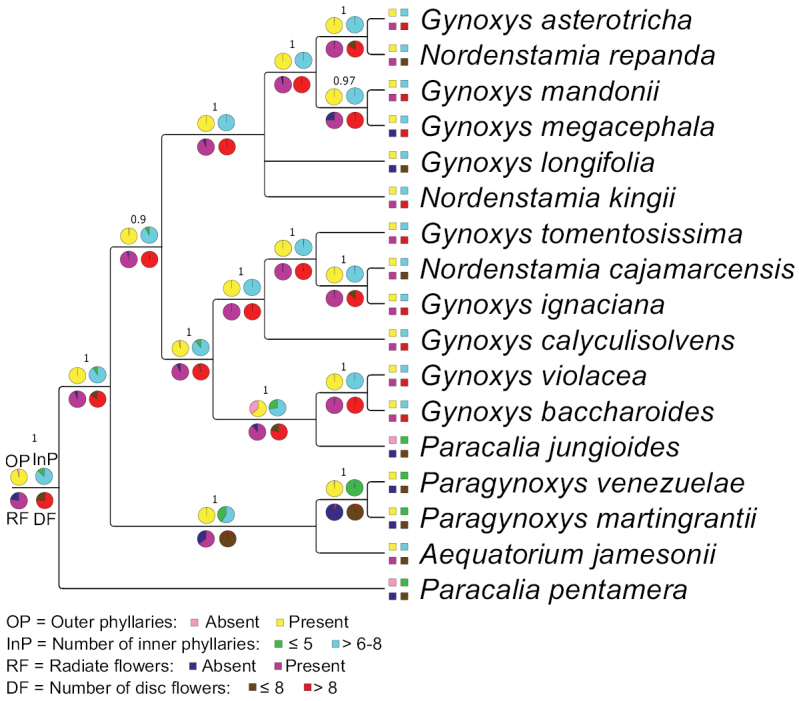
Bayesian inference of ancestral character state reconstruction of four morphological characters of the Gynoxyoid clade in the consensus plastome tree by Escobari et al. (2021). Each pie chart represents a single character and each colour represents a character state which is described in the legend. The actual state of the characters is represented by boxes next to the species names. The pie charts at the stem of the tree show the character abbreviations as mentioned in Table 1.

**Figure 4.**
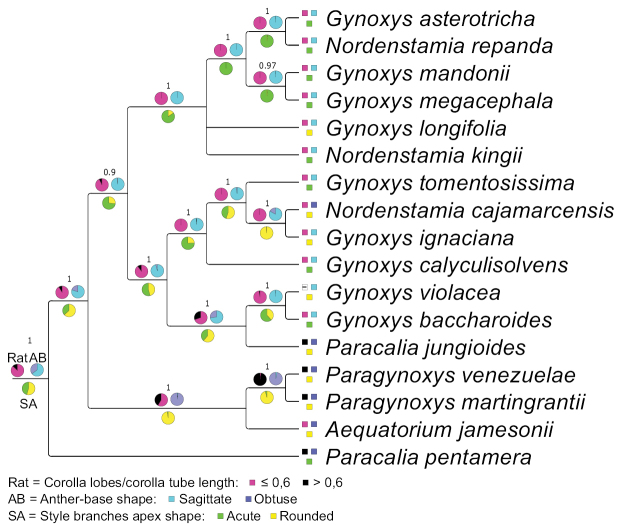
Bayesian inference of ancestral character state reconstruction of four morphological characters of the Gynoxyoid clade in consensus plastome tree by Escobari et al. (2021). Each pie chart represents a single character and each colour represents a character state which is described in the legend. The actual state of the characters is represented by boxes next to the species names. The pie charts at the stem of the tree show the character abbreviations as mentioned in Table 1. Missing data are represented as (-).

Employing the eleven characters of Table 1, a species-based matrix was created for the 17 members of the Gynoxyoid clade represented in the phylogenetic tree by Escobari et al. (2021) (Table 2) and used for ancestral character reconstruction. Two character states for *G.ignaciana* (colour of flowers) and *G.violacea* (length ratio disc flower corolla lobe/tube) were coded as missing because they were not accessible in the material at hand. The accession *Gynoxys* sp. in Escobari et al. (2021) was identified as *G.calyculisolvens* during this study. The ancestral character reconstructions for the eleven characters in the Gynoxyoid clade are presented in Figs 2–4.

The Gynoxyoids exhibit various evolutionary changes in their characteristics. The shrubby habit was initially considered primitive, but two independent shifts to a scandent habit occurred in the two *Paracalia* species, while a shift from shrub to tree habit was observed in *Paragynoxys* and within the *Gynoxys* clade. Opposite phyllotaxis was revealed as the ancestral state, but shifts to alternate phyllotaxis occurred in *Paracaliapentamera*, the stem node of *Paragynoxys*, two (out of three) species of *Nordenstamia* and *Paracaliajungioides*. Unicellular trichomes were revealed as ancestral for all Gynoxyoids, but *Paracaliapentamera* became glabrous. Multicellular hairs emerged in the most recent common ancestor of *Aequatorium* and *Paragynoxys*, as well as in certain species within the *Gynoxys* clade. White flowers were revealed as the ancestral state, retained by the earliest diverging clades (*Aequatorium*, *Paragynoxys* and *Paracaliapentamera*), while yellow flowers appeared at the stem node of *Gynoxys* and *Nordenstamia*. A reversal to white flowers occurred in *Paracaliajungioides*, nested within the *Gynoxys* clade. A higher number of inner phyllaries was ancestral, but both *Paracalia* and *Paragynoxys* species showed a decrease in this number. Radiate flowers were ancestral, but discoid capitula emerged in all *Paracalia* and *Paragynoxys* species, with additional losses of ray flowers in some *Gynoxys* species. A high number of disc flowers was the ancestral state, but reductions occurred at the stem node of *Aequatorium* and *Paragynoxys* and in all *Paracalia* species, partially within the *Gynoxys* clade. A shallow division of the corolla into lobes was revealed as plesiomorphic and retained in *Aequatorium* and all *Gynoxys*, but changed in *Paracalia* and *Paragynoxys* to a deep division. The style branch apex was rounded ancestrally, retained in *Aequatorium* and *Paragynoxys*, but an acute apex appeared in the earliest diverging species, *Paracaliapentamera*, with further shifts and reversals in *Gynoxys*, *Nordenstamia* and *Paracaliajungioides*.

**Figure 5.**
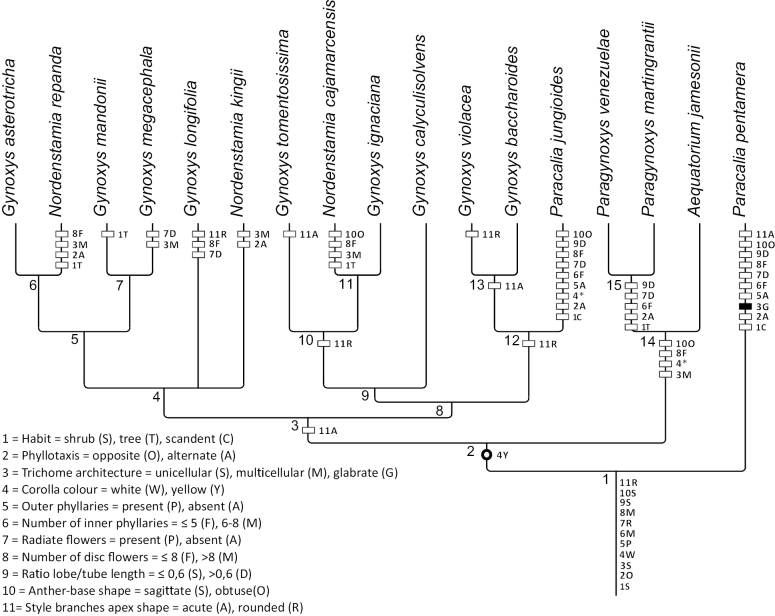
Summary tree based on the results of the BayesTraits analysis (Figs 2–4) of state shifts in morphological character. A threshold of 0.75 was used to define the character shifts between states. Characters with multiple state shifts (homoplasies) are shown with white boxes, reversals are indicated by * and unresolved shifts are indicated by an open circle. Numbers at the left of the branches represent the nodes in Suppl. material 3.

A summary of the BayesTraits analysis of all state shifts for each character in Figs 2–4 is given in Fig. 5. Characters are represented by numbers and states with the codes given in Table 1. A high number of shifts occur in the two species of *Paracalia* because the genus is retrieved as non-monophyletic in the plastid topology, although its species share most morphological character states. The clade represented by both *Paragynoxys* species shared all derived characters with *Aequatoriumjamesonii* in addition to five derived characters that characterise the clade. A single character (corolla colour) was retrieved as synapomorphic for the clade containing *Aequatorium*, *Gynoxys*, *Paragynoxys*, *Nordenstamia* and *Paracaliajungioides* and even this character shows several reversals at the MCRA of *Aequatorium* and *Paragynoxys* and of both species of *Paracalia*. The analysis retrieved most of the morphological characters as highly homoplastic with the style branch apices being the most variable character throughout the tree at many nodes. A summary of the BayesTraits analysis with each character at each node is given in Suppl. material 3.

### ﻿Checklist of the *Gynoxys* clade

The initial revision of the different databases resulted in a variable number of species. The Compositae Global Database lists 270 names (May 2023), The World Flora online includes 257 (Dec 2022), IPNI registers 242 (May 2023) and Tropicos has 243. This study resulted in a checklist comprising a total of 209 names of which 158 are accepted. Additionally, eleven names were newly synonymised, two were lectotypified and eight were newly transferred to other genera.

## ﻿Discussion

### ﻿Trees inferred from plastid genomes and putative cytonuclear discordance

In the present investigation, we attempted to compare, for the members of the Gynoxyoid clade, tree reconstructions, based on the plastid genome and on the ITS and ETS nuclear ribosomal regions. Variation amongst the plastid genomes was extremely low (Escobari et al. 2021) and even more so in the nuclear ribosomal DNA. The lack of resolution in the nuclear ribosomal trees severely limits the comparison of phylogenetic signal from the organellar and nuclear genomic compartments. Nevertheless, there are some noteworthy exceptions. One is the unequivocal support for the sister group relationship of the two *Paragynoxys* members. The second is the missing support for the monophyly of the three *Nordenstamia* members in all three trees. The most significant result is, however, the gene tree incongruence concerning the two *Paracalia* species. The plastid tree placed *Paracaliajungioides* within *Gynoxys* and far distant from the second species, *P.pentamera* (Escobari et al. 2021). In contrast, the two species of *Paracalia* are supported as monophyletic in the ETS tree in conformity with morphology, although not in the ITS nor in the concatenated ETS-ITS tree (Suppl. material 2: appendix 2d). This finding is surprising because *Paracaliajungioides* is morphologically very distinct from all members of *Gynoxys.* It is scandent (instead of a tree or shrub), has white (instead of yellow) flowers and an involucre without outer phyllaries (instead of present). Moreover, *P.jungioides* and *P.pentamera* are morphologically very similar and the plastome phylogeny would suggest that these species have accumulated a high number of independent parallel state shifts (i.e. scandent habit, alternate leaves, absent outer phyllaries, few inner phyllaries, discoid capitula, few number of disc flowers, deep-lobed corolla, obtuse anther base) (Figs 3, 5). We assume that the incongruence with respect to the position of the two *Paracalia* species between the morphological data and the ETS topology on the one hand and the plastome tree topology on the other hand, is best explained by a chloroplast capture event. Chloroplast capture occurs when two species hybridise and go through extensive backcrossing to one of the ancestors (Rieseberg and Soltis 1991). The hybridisation event followed by extensive backcrossing swamp out the nuclear signal, but the captured plastid remains (Kandziora et al. 2022). In our case, we assume that *P.jungioides*, after introgression with a *Gynoxys* species, has captured the plastome of a member of the latter genus.

Nuclear-cytoplasmic incongruences have been reported in several studies within the Asteraceae family at higher and specific levels (Kilian et al. 2017; Pascual-Díaz et al. 2021; Senderowicz et al. 2021), especially in the Senecioneae (Pelser et al. 2007, 2010). It has also been shown by Stull et al. (2020) for the asterids that conflicts between nuclear and plastome trees are a relevant issue even at higher evolutionary scales. Phylogenetic inferences on nuclear data recovered different placements for several asterid lineages compared to topologies on plastid data (Yin et al. 2021; Kandziora et al. 2022). This is of some significance when we consider that current backbones of angiosperm phylogeny are largely based on plastid phylogenies (APG IV 2016). Amongst the principal reasons for these incongruences, horizontal gene flow amongst lineages, introgression, hybridisation and incomplete lineage sorting were suggested (Rieseberg and Soltis 1991; Maddison 1997; Vargas et al. 2017). The inclusion of different markers of different origins in a phylogenetic analysis has the capacity to elucidate signals of such events. Pelser et al. (2010) analysed potential causes for tree incongruences in the tribe Senecioneae comparing two nuclear (ITS/ETS) and six plastid markers. They concluded that hybridisation is a much more likely explanation than ILS, long-branch attraction or sampling error. Lee-Yaw et al. (2018) focused their study on organelle discordances by sequencing whole plastomes and over 1000 nuclear single-nucleotide polymorphisms in *Helianthus* L. The authors showed that incongruences in this genus can be expected at species level and amongst individuals of the same species. The Gynoxyoid clade is a further example of short molecular distances on plastid and ribosomal markers amongst species. The lack of molecular variability hampers the reconstruction of well-supported clades on this type of data; nevertheless, the great morphological variation enables the definition of morpho-species in many cases. On the other hand, the phylogenetic reconstruction, although with moderate support, can give evidence to support the assignment of morphologically similar individuals to the same entities (i.e. hypothesised species).

Gene tree discordance is expected to be more likely in rapid radiating lineages that can be found in young biodiversity hotspots, such as the Andean Region (Madriñán et al. 2013; Kandziora et al. 2022). The fast succession and accumulation of descendant species are prone to inter-breeding before reproductive barriers develop, increasing the probability of incomplete lineage sorting (ILS) (Vargas et al. 2017). In addition, young radiating groups have shown whole genome duplication and hybridisation events in the tropical high-altitude areas of South America (*Lachemilla*: Morales-Briones et al. 2018; *Lupinus*: Nevado et al. 2018; *Diplostephium*: Vargas et al. 2017; Espeletiinae: Cortés et al. 2018). Hybridisation may be a result of sexual selection, ecological adaptation, pollinator changes (Moreira-Munoz 2020; Kandziora et al. 2022) or due to the dynamic changes in habitat connectivity in this ecosystem with multiple topography changes during the Pleistocene (Flantua et al. 2019) which facilitated the contact between geographically isolated species before exhibiting strong barriers to gene flow (Vargas et al. 2017; Kandziora et al. 2022). Vargas et al. (2017) revealed complex patterns of reticulate evolution at generic and species level of *Diplostephium*.

### ﻿Evolution and significance of morphological characters in the Gynoxys clade

Previous generic classifications of the Gynoxyoid group were based on morphological similarities and discontinuities between species assemblages. In this study, we tested these hypotheses by optimising character states on the full plastome phylogeny (Escobari et al. 2021, see also Suppl. material 2: appendix 2a). Morphological differentiation amongst the Gynoxyoids is shallow and limited to comparatively few and often rather subtle characters. The most recent ancestor of the Gynoxyoid had a shrubby habit, opposite leaves and it was vested by unicellular simple hairs. The capitula was radiate, equipped with outer phyllaries and 6–8 inner phyllaries and had up to eight disc flowers. The corolla was whitish and the corolla lobes were remarkably shorter than the corolla tube. Most of these plesiomorphic states (except the whitish corolla) were retained by most of the species of the genus *Gynoxys* during its evolution. Shifts in the character states are evident in the rest of the Gynoxyoid members. Specially, the switch from whitish to yellowish corolla (which is apparently the only synapomorphy under the given tree inference) resulted as unresolved due to a small difference of the PP values (59% yellow vs. 41% white). All shifts reconstructed under the ancestral character reconstruction were retrieved as highly homoplasious and are, therefore, unsuitable for genera characterisation under the given plastid inference presented in Escobari et al. (2021).

### ﻿Species diversity of the Gynoxyoid clade

Our taxonomic backbone provides the best estimate of species diversity in the Gynoxyoid clade. Type information has been synthesised here for the first time in a comprehensive way. Further taxonomic knowledge turnover is expected at species level once species limits are tested in an integrative approach in a separate paper. Specially the examination of a reduced group of Bolivian species depicted shallow morphological differences, making the taxonomy complicated and predicting further nomenclatural changes. Additionally, the low number of collections available hinders a full examination of the species limits.

### ﻿Taxonomic conclusions

#### ﻿Gynoxyoid clade

158 species

Argentina, Bolivia, Colombia, Ecuador, Peru, Venezuela

Trees, shrubs or scandent vines. Indumentum tomentose, of unicellular (simple) or multicellular trichomes (simple, stellate, T-shaped, multibranched), becoming rusty or greyish-white with age on petioles, abaxial side of the leaves and involucres. Leaves alternate, opposite or subopposite, petiolate or subsessile; margin angulate, dentate, denticulate, entire, sinuate or repand, callous-tipped teeth present or absent; base acute, cordate, cuneate, obtuse, oblique, rotund or truncate; apex acute, acuminate, attenuate, mucronate, obtuse or rotund; coriaceous or papyraceous; leaf indumentum on abaxial leaf surfaces rusty-brownish or greyish-white with age. Synflorescence terminal, subterminal or axillary, thyrsoidiform, paniculiform or corymbiform, peduncles bracteolate. Capitula heterogamous or homogamous, numerous, Receptacle flat to convex. Involucre campanular or tubular; outer phyllaries 0–8; inner phyllaries 5–10 (–13), uni- or biseriate. Ray flowers 0–8 (–13), female; tube cylindrical, glabrous; ligule white, cream-coloured or yellow, almost equalling the tube in length, 3–4–veined, 3-toothed at the apex, with a papillate upper surface. Style bifid, fertile. Disc flowers 5–32 (–36), hermaphrodite; corolla campanulate or funnel-shaped, white, pale greenish-yellow or yellow, shortly or largely lobed; lobes ovate, triangular or oblong, straight, recurved to the outside or helically twisted. Anthers exserted; apical appendage oblong-ovate or obtuse; base obtuse, auriculate or sagittate; filament collar narrowly cylindrical, uniform or thicker than the filament; with polar endothecial thickenings (Jeffrey 1992). Style-base gradually dilated, placed on a nectary; style branches straight or contorted, apically obtuse, truncate or acute, with papilliform sweeping-hairs. Achenes homomorphic, oblong, glabrate, ribbed. Pappus bristles pluriseriate, persistent, coarse, shortly barbellate, off-white or somewhat brownish-fulvous. *n* = ca. 40 (Watanabe 2002).

### ﻿Key to the genera of the Gynoxyoid clade

**Table:** 

1a	Flowers white	**2**
2a	Leaves, stems (in young shoots) and involucres with peltate-stellate hairs; capitula radiate	***Aequatorium***
2b	Leaves, stems (in young shoots) and involucres glabrous, with single or stellate hairs; capitula discoid	**3**
3a	Trees; leaves, stems (in young shoots) and involucres with stellate hairs; outer phyllaries present. Colombia and Venezuela	***Paragynoxys***
3b	Scandent shrublets; leaves, stems (in young shoots) and involucres with hairs absent or simple; outer phyllaries absent. Bolivia and Peru	***Paracalia***
1b	Flowers yellow	***Gynoxys***

### ﻿Revised classification of the genera and species of the Gynoxyoid clade

**1. *Aequatorium* B.Nord. in Opera Bot. 44: 59. 1978 (Fig. 1f)**

Type: *Aequatoriumasterotrichum* B.Nord.

12 species

Colombia, Ecuador

Erect shrubs or trees, sometimes with sub-scandent branches. Indumentum tomentose, of subsessile stellate trichomes (with 1–3-tiered, irregularly star-shaped, subtended by a narrow pluricellular uniseriate stalk), with age, glabrescent, but with persistent greyish-white tomentum on petioles, abaxial side of leaves and sometimes involucres. Leaves alternate or subopposite or rarely opposite, petiolate, rounded-elliptic to lanceolate; margin entire, sinuate-dentate or denticulate, with small callous-tipped teeth; base acute, cuneate to rounded-truncate, subcordate or oblique; apex acute or rotund; coriaceous; leaves indumentum rusty-brownish on the adaxial side with two layers of peltate-stellate hairs, internal layer with sessile hairs and outer layer with subsessile hairs in patches, becoming grey-tomentose with age. Synflorescence terminal, rarely subterminal, (thyrsoid-) paniculiform or corymbiform. Capitula heterogamous. Receptacle flat or slightly convex. Involucre campanular; outer phyllaries usually < 6; inner phyllaries 5–10, biseriate. Ray flowers usually < 5 (–8); ligule white or cream-coloured. Disc flowers 5–10; corolla campanulate or funnel-shaped, white or pale greenish-yellow, shortly lobed, ratio lobes/tube < 0.8; lobes narrowly ovate, triangular or oblong, recurved to the outside or straight. Anther base sagittate-auriculate; filament collar narrowly cylindrical, uniform, not thicker than the filament. Style branches half contorted, apically obtuse or truncate. Distribution: Colombia, Ecuador.

Notes: We exclude *Aequatoriumvenezuelanum* from this genus, based on its yellow flowers and distribution and transfer this species to *Gynoxys*.

***Aequatoriumalbiflorum*** (Wedd.) Cuatrec. & S.Díaz, Revista Acad. Colomb. Ci. Exact. 17(67): 665. 1990 ≡ *Gynoxysalbiflora* Wedd., Chlor. Andina 1(3): 78. 1856 [“1855”]. – Syntypes: Colombia. Mariquita, sur la lisière du volcan de Tolima, 3900 m, Jan 1843, *J. Linden 907* (F: V0076792FV0076793F (photo & fragments), K: K000497659, NY 178788, P: P00711390P00711391P02273078).

***Aequatoriumasterotrichum*** B.Nord., Opera Bot. 44: 59. 1978. – Holotype: Ecuador. Pichincha, lago Papallacta, thicket, 3300 m, 31 Oct 1955, *E. Asplund 18263* (S: S-R–8297; isotypes: k: K000497658, LD 1821970, MO: MO–3237504, NY, P: P00971087, R, S: S18–7665, UPS, US).

***Aequatoriumcaucanum*** S.Díaz & Cuatrec., Revista Acab. Colomb. Ci. Exact. 73: 248. 1994. – Holotype: Colombia. Cauca, Macizo Central Colombiano, Páramo de las Papas, El Boquerón, 3200–3510 m, 7–27 Sep 1958, *J. Idrobo et al. 3221* (COL: COL000004758).

= Aequatoriumcaucanumvar.abbreviatum S.Díaz & Cuatrec., Revista Acab. Colomb. Ci. Exact. 73: 248, f. 2. 1994. – Holotype: Colombia. Cauca, Volcán Puracé, alrededores de la Laguna San Rafael, 3340 m, 6 Jan 1972, *A. M. Cleef & A. Fernandez 526* (COL: COL000004759).

***Aequatoriumjamesonii*** (S.F.Blake) C.Jeffrey, Kew Bull. 47(1): 61. 1992 ≡ *Gynoxysjamesonii* S.F.Blake, Acad. Sci. 18: 34. 1928. – Holotype: Ecuador. Pichincha, west side of Mount Pichincha, 3050 m, 2 Aug 1926, *Jameson 227* (K: K000497657; isotype: US 00122911 (fragments & photo)).

= *Seneciosimulans* Benoist, Bull. Soc. Bot. France 83: 808. 1937, nom. illeg. [non *Seneciosimulans* Chiov. 1935] ≡ *Gynoxyssimulans* Cuatrec., Brittonia 8: 158. 1955. – Syntype: Ecuador. Pichincha, 12 Jul 1931, *Benoist 4572* (P: P02273075).

***Aequatoriumlatibracteolatum*** S.Díaz & Cuatrec., Revista Acad. Colomb. Ci. Exact. 17(67): 661, 663, f. 1. 1990. – Holotype: Colombia. Cauca, Municipio de Puracé, Parque Nacional Natural del Puracé, cercanías de la Laguna San Rafael, 3300 m, 6 Oct 1984, *C. G. Lozano 4667* (COL: COL000004762; isotypes: COL: COL000004760COL000004761).

***Aequatoriumlepidotum*** B.Nord., Compositae Newslett. 31: 6, f. 3. 6B. 1997. – Holotype: Ecuador. Carchi, El Mirador, 15 km S of San Francisco, 00°37‘N, 77°31‘W, 3300 m, 2 Aug 1990, *W. Palacios & D. Rubio 5286* (MO: MO–037535; isotype: US 01919680).

***Aequatoriumpalealbum*** S.Díaz & A.Correa, Revista Acad. Colomb. Ci. Exact. 26(100): 345–346, f. 3. 2002. – Holotype: Colombia. Nariño, Ospina, páramo de Paja Blanca, alrededores de la bocatoma del acueducto, 1°58‘N, 77°34‘W, 3200 m, 2 Dic 1995, *B. Ramirez-P. et al. 8904* (PSO: PSO0000058; isotype: MO: s.n.).

***Aequatoriumpolygonoides*** B.Nord., Opera Bot. 44: 63. 1978 ≡ *Seneciopolygonoides* Cuatrec., Notas a la Flora de Colombia 6: 20, f. 14. 1944, nom. illeg. [non *Seneciopolygonoides* Muschl. 1911]. – Holotype: Colombia. Caldas, Cordillera Central, vertiente occidental, vertiente SE del Nevado del Ruiz, Termales, 3400 m, 4 May 1940, *J. Cuatrecasas 9243* (COL).

***Aequatoriumrepandiforme*** B.Nord., Compositae Newslett. 31: 9, f. 4. 1997. – Holotype: Ecuador. Pichincha, over high pass en route to Quito, 92 km E of Quevedo, 3400 m, 19 Sept 1959, *B. Maguire & C. Maguire 44246* (NY 3468431; isotypes: K: K000497656, US 01919679).

***Aequatoriumsinuatifolium*** S.Díaz & A.Correa, Revista Acad. Colomb. Ci. Exact. 19(73): 251–252, f. 3. 1994. – Holotype: Colombia. Quindío, Mun. de Salento, arriba de Guayaquil, 3680 m, 10 Jan 1994, *W. G. Vargas 1335* (COL: COL000004764; isotype: COL: COL000004763).

***Aequatoriumtatamanum*** S.Díaz & A.Correa, Revista Acad. Colomb. Ci. Exact. 23(88): 332, f. 1. 1999. – Holotype: Colombia. Risaralda, Municipio de Santua Río, Macizo de Tatamá, 200 m arriba del campamento El Reposo, 3700 m, 8 Feb 1983, *J. H. Torres et al. 1720* (COL: COL000004765).

Note: This species is probably conspecific with *A.albiflorum*.

***Aequatoriumverrucosum*** (Wedd.) S.Díaz & Cuatrec., Revista Acad. Colomb. Ci. Exact. 17(67): 659–666. 1990 ≡ *Gynoxysverrucosa* Wedd., Chlor. Andina 1(3): 77. 1856 [“1855”] [*non Gynoxysverrucosa* V.M.Badillo 1946]. – Lectotype (Diaz & Cuatrecasas 1990: 663): Colombia. Nueva Granada, Mariquita, Cordillere de Quindiu, a Los Volcancitos, 3200 m, Jan 1843, *Linden 1050* (P: P02273077; isolectotypes: F: V0076796FV0076797FV0076798F, K: K000497655).

= *Senecioverrucosus* Klatt, Abh. Naturf. Ges. Halle 15(2): 332. 1881 [1882]. –Syntypes: *Triana s.n.* (P; photo: F).

**2. *Paracalia* Cuatrec., Brittonia 12: 183. 1960 (Fig. 1b, e)**

Type: *Paracaliapentamera* (Cuatrec.) Cuatrec.

2 species

Bolivia, Peru

Scandent shrublets. Indumentum glabrate to glabrescent, of simple hairs, glabrate with age. Leaves alternate, petiolate, ovate; margin entire or angulate, with or without small callous-tipped teeth; base rotund or cordate; apex acuminate or mucronate; coriaceous; leaves glabrous or pilose on the adaxial site, but glabrescent with age. Synflorescence terminal or axillar, paniculiform or corymbiform. Capitula homogamous. Receptacle flat. Involucre tubular; outer phyllaries absent; inner phyllaries 5, uniseriate. Ray flowers absent. Disc flowers 5; corolla campanulate, white or pale greenish, deeply lobed, ratio lobes/tube ≈ 1; lobes linear, helically twisted. Anther base auriculate or obtuse; filament collar cylindrical, thicker than the filament. Style branches half contorted, apically obtuse or subtruncate. Distribution: Peru, Bolivia.

Note: Although the phylogenetic inferences suggest this genus to be not monophyletic, we kept the circumscription of *Paracalia* including two species. We substantiate this decision based on shared morphological characters, such as deeply lobed and white-flowered corolla and the central Andean distribution beginning from lowlands (800 m). *Paracaliajungioides* which is nested in the *Gynoxys* clade strikingly differs morphologically from the true *Gynoxys* species and its inclusion in this genus would break the continuity of the morphological characters and altitudinal distribution in this group. A possible explanation for the contradiction between morphological/ecological and molecular data may be chloroplast capture and this needs to be further studied and better understood before further nomenclatural decisions are made. In this context, we think the best practice is to retain the current circumscription of *Paracalia* and avoid suggesting further possibly wrong hypotheses of relationships of these species.

***Paracaliajungioides*** (Hook. & Arn.) Cuatrec., Brittonia 12: 183. 1960 ≡ *Pentanthusjungioides* Hook. & Arn., Companion Bot. Mag. 1: 33. 1835. – Holotype: Perú. Purruchuca, Jun 1833, *Matthews 1016* (K: K000497546(!); isotypes: E: E00414051E00414052, K: K000497547(!)).

= *Cacaliamikaniifolia* DC., Prodr. 6: 328. 1837 ≡ *Seneciomikaniifolius* (DC.) Sch.Bip., Flora 28: 498. 1845. – Syntypes: Peru. San Buenaventura, *Nee & Thibaud s.n.* (not traced in G-DC, F: s.n. (photo)).

***Paracaliapentamera*** (Cuatrec.) Cuatrec., Brittonia 12: 183. 1960 ≡ *Seneciopentamerus* Cuatrec., Fieldiana, Bot. 27: 57. 1951. – Holotype: Bolivia. La Paz, Larecaja, Copacabana (ca. 10 km. south of Mapiri), 850–950 m, 08 Oct – 15 Nov 1939, *B. A. Krukoff 11150* (NY 259336(!); isotypes: A: A00010877, F: V0077069F, K: K000497545(!), S: S-R–7986, U 0105750, US 00123446).

**3. *Paragynoxys* (Cuatrec.) Cuatrec., Brittonia 8: 153. 1955. (Fig. 1b, c)**

≡ Seneciosect.Paragynoxys Cuatrec., Fieldiana, Bot. 27(2): 72. 1951.

Type: *Paragynoxysneodrendoides* (Cuatrec.) Cuatrec.

13 species

Colombia, Venezuela

Erect shrubs or trees. Indumentum tomentose of (always?) stellate T-shaped trichomes, persistent in all age states, becoming greyish-white on petioles, abaxial side of leaves and involucres. Leaves alternate or rarely opposite, petiolate, oblong-elliptic, obovate-elliptic or ovate; margin entire or repand, with or without small callous-tipped teeth; base cordate, obtuse or rarely cuneate; apex obtuse, attenuate or rarely acute; coriaceous; leaf indumentum shaggy rusty-brownish in the adaxial site, persistent with age. Synflorescence terminal rarely subterminal, (thyrsoid-) paniculiform or corymbiform. Capitula homogamous. Receptacle flat. Involucre campanular; outer phyllaries < 6; inner phyllaries 5 or 8, uniseriate. Ray flowers absent. Disc flowers 5–11; corolla campanulate, white, deeply lobed, ratio lobes/tube => 1; lobes linear, helically twisted. Anther base auricular or obtuse; filament collar cylindrical, thicker than the filament. Style branches fully contorted (forming a complete loop or even two), apically obtuse to subacute. Distribution: Colombia, Venezuela.

Note: We support the view of Correa (2003) who transferred *Paragynoxysregis* back to *Gynoxys* (as it was originally described), based on its radiate capitula with yellow flowers and distribution.

***Paragynoxysangosturae*** (Cuatrec.) Cuatrec., Brittonia 8: 154. 1955 ≡ *Senecioangosturae* Cuatrec., Feddes Repert. Spec. Nov. Regni Veg. 55: 132. 1953. – Holotype: Colombia. Antioquia, Angostura, just outside town, 2000 m, 11 Mar 1944, *F. R. Fosberg 21603* (US 00123252; isotypes: US 0012325300123254).

***Paragynoxyscorei*** (Cuatrec.) Cuatrec., Brittonia 8: 154. 1955 ≡ *Seneciocorei* Cuatrec., Feddes Repert. Spec. Nov. Regni Veg. 55: 136. 1953. – Holotype: Colombia. Antioquia, Alto El Oso, n. of Yarumal, 2320 m, 4 Mar 1944, *E. L. Core 624* (F: V0051295F; isotype: US 00123277).

***Paragynoxyscuatrecasasii*** Ruiz-Teran & López-Fig., Revista Fac. Farm. Univ. Andes 14: 14, f. 3, 4. 1974. – Holotype: Venezuela. Merida, Rangel, norte de la población Las Piedras, Cuenca del río Aracay, afluente del Santo Domingo, 2550–2700 m, 16 Dic 1972, *Ruiz-Terán et al. 8258* (MERF; isotype: US).

***Paragynoxysmagnifolia*** Cuatrec., Brittonia 8(2): 154. 1955. – Holotype: Venezuela. Merida, Culata, 7000 ft., May 1847, *N. Funck & Schlimm 1522* (P: P00711443P00711444; isotypes: G: G00301285, P: P00711445P00711446, US 00811048, VEN: VEN118056 (fragments of holotype)).

***Paragynoxysmartingrantii*** (Cuatrec.) Cuatrec., Brittonia 8: 156. 1955 ≡ *Seneciomartingrantii* Cuatrec., Feddes Repert. Spec. Nov. Regni Veg. 55: 139. 1953. – Holotype: Colombia. Magdalena, Sierra de Perijá, Casacará Valley 23 km. East of Codazzi, 2 km from the Venezuelan border, 2450, 15 Feb 1945, *M. L. Grant 10949* (F: V0051336FV0051337F; isotypes: COL: COL000005419, HUA: HUA0000364, NY 259292259293, US 0012332400123323, VEN: VEN209193, WIS: WISv0256984WISWISv0256985WIS).

***Paragynoxysmeridana*** (Cuatrec.) Cuatrec., Brittonia 8(2): 156. 1955 ≡ *Gynoxysverrucosa* V.M.Badillo, Bol. Soc. Venez. Ci. Nat. 10: 312. 1946, nom. illeg. [*non Gynoxys verrucosa* Wedd. 1855] ≡ *Seneciosteyermarkii* Cuatrec., Fieldiana, Bot. 27: 32–33. Jun 1950, nom. illeg. [non *Seneciosteyermarkii* Greenm. Apr 1950] ≡ *Seneciomeridanus* Cuatrec., Fieldiana, Bot. 27(2): 38. 1951. – Syntypes: Venezuela. Merida, Paramo de Pozo Negro between San José and Beguilla, 2590–3220 m, 3 May 1944, *A. Steyermark 56268* (NY 259418, US 00123361).

***Paragynoxysneodrendoides*** (Cuatrec.) Cuatrec., Brittonia 8(2): 156, f. 13, 14. 1955 ≡ *Senecioneodendroides* Cuatrec., Notas Fl. Colombia 6: 19, f. 13. 14. 1944. – Holotype: Colombia. Santander, Cordillera Oriental, Páramo de la Rusia, vertiente noroeste, 3300–3500 m, 4 Ago 1940, *J. Cuatrecasas 10435* (COL; isotypes: F: V0051343FV0051344FV0051345F, P: P01816686).

***Paragynoxyspileolanata*** S.Díaz, Caldasia 12(59): 379–381, f. 1. 1979. – Holotype: Colombia. Santander, Municipio de Onzaga, vereda Chaguaz, finca de Oliverio Mesa, en robledal, 2820 m, 29 Mar 1976, *J. H. Torres et al. 500* (COL: COL000005310).

***Paragynoxyssanturbanensis*** (Cuatrec.) Cuatrec., Brittonia 8(2): 156. 1955 ≡ *Seneciosanturbanensis* Cuatrec., Feddes Repert. Spec. Nov. Regni Veg. 55: 145. 1953. – Holotype: Colombia. Santander, Páramo de Santurbán, vert. W, 3100 m, 27 Jul 1940, *J. Cuatrecasas & H. García Barriga 10326* (F: V0051361FV0051362F; isotype: P: P01816508).

***Paragynoxyssteyermarkii*** Cuatrec., Phytologia 40(1): 34. 1978. – Holotype: Venezuela. Táchira, Between Las Copas de Alto de Fila de Tierra Negra at the ridge dividing headwaters of rivers Quinimarí, Riofrio, Uribante and Talco (Oirá), 2870–2880 m, 16 Jan 1968, *J. A. Steyermark & E. Dunsterville 101014* (US 00115958; isotypes: MA 638740, US 00115959, VEN: VEN74042).

***Paragynoxysundatifolia*** Cuatrec., Proc. Biol. Soc. Washington 74: 15. 1961. – Holotype: Colombia. Magdalena, Sierra Nevada de Santa Marta. Southeastern slope: Hoya del Río Donachuí, below Sabanita Diricune, 3200 m, 29 Sep 1959, *J. Cuatrecasas & R. Romero-Castañeda 24485* (US: US00115961 US00115962 US00115963 US00115964 US00115965; isotypes: COL: COL000005311COL000005312COL000005313COL000005314, P: P00711449P00711450, US: US00115960 US00930968).

***Paragynoxysuribei*** Cuatrec., Phytologia 40(1): 33. 1978. – Holotype: Colombia. Boyaca, Arcabuco, 4 km. NE of town, 08 Jun 1966, *L. U. Uribe 5633* (US: US00115966 US00115967; isotypes: COL: COL000005315COL000005316).

***Paragynoxysvenezuelae*** (V.M.Badillo) Cuatrec., Brittonia 8(2): 156. 1955 ≡ *Cacaliavenezuelae* V.M.Badillo, Bol. Soc. Venez. Ci. Nat. 10: 319. 1947 ≡ *Seneciovenezuelae* (V.M.Badillo) Cuatrec., Fieldiana, Bot. 27(1): 31. 1950. – Holotype: Venezuela. Merida, Below páramo above San Isidro Alto, 1820 m, 14 May 1944, *J. Steyermark 56560* (VEN: VEN32772; isotypes: F: V0049135F, NY 162855).

**4. *Gynoxys* Cass. in Cuvier, Dict. Sci. Nat., ed. 2, 48(2): 455. 1827. (Fig. 1a, c–f)**

Lectotype (Flann et al. 2010: 1225): *Gynoxysbaccharoides* (Kunth) Cass.

= *Nordenstamia* Lundin, Compositae Newslett. 44: 15–16, f. 1. 2006, syn. nov. Type: *Nordenstamiarepanda* (Wedd.) Lundin [≡ *Gynoxysrepanda* Wedd.]

130 species

Argentina, Bolivia, Colombia, Ecuador, Peru, Venezuela

Erect shrubs or trees. Indumentum absent or tomentose, of simple or multicellular simple trichomes, glabrescent with age, but with persistent greyish-white tomentum on abaxial side of all leaves and involucres. Leaves alternate or opposite, petiolate or subsessile, elliptic, lanceolate, ovate, obovate; margin entire, repand, sinuate, sparsely angular or denticulate, with small callous-tipped teeth; base acute, attenuate, cordate, cuneate, obtuse, rotund, truncate or oblique; apex acute, acuminate, obtuse or mucronate; coriaceous or papyraceous; leaf indumentum absent, white or rusty-brownish in the adaxial site, persistent with age. Synflorescence terminal or axillar, paniculiform or corymbiform. Capitula homogamous (Sec 1) or heterogamous. Receptacle flat or convex. Involucre campanular; outer phyllaries 1–8; inner phyllaries usually 5–8(–13), biseriate. Ray flowers usually < 8 (–10–13); ligule yellow. Disc flowers usually 5–32 (–36); corolla tubular, campanulate or funnel-shaped, yellow, usually shortly lobed, ratio lobes/tube usually < 0.5; lobes triangular, oblong or narrowly ovate, recurved to the outside or straight. Anther base sagittate, auriculate or rarely obtuse; filament collar narrowly cylindrical, usually thicker than the filament. Style branches straight or half contorted, apically obtuse, truncate or acute.

Note: *Gynoxysalternifolia* and *G.mandonii* were described in literature as scandent. This information is certainly erroneous; in the field, we had a chance to trace several individuals of *G.mandonii* as large trees with thick branches and the type specimen of *G.alternifolia* also shows thick and erect branches with no sign of a liana-like growth.

**Table:** 

1a	Capitula discoid	***Gynoxys* , discoid group**
1b	Capitula radiate	**2**
2a	Leaves, stems (in young shoots) and involucres with stellate hairs	***Gynoxys* , *Praegynoxys* group**
2b	Leaves, stems (in young shoots) and involucres with simple hairs	***Gynoxys* , *Gynoxys* s.str. group**

***Gynoxyscampii*** Cuatrec., Brittonia 8(1): 39. 1954. – Holotype: Ecuador. Cañar, Near El Tambo (ca. 69 km. by railroad south of Sibambe), 9500–10000 ft., 5 Jul 1945, *W. H. Camp E–3970* (F: V0076704F; isotypes: G: G00223899, GH: GH00008575, K: K000497540, NY 178793, P: P00711407, US 00122893, VEN: VEN34418).

***Gynoxysdielsiana*** Domke, Biblioth. Bot. 116: 169. 1937. – Syntype: Ecuador. Chimborazo, Tipococha, untere Rand des Paramo, ca. 3230 m, 20 Aug 1933, *L. Diels 675* (B, destroyed).

***Gynoxyshutchisonii*** H.Rob. & Cuatrec., Novon 2(4): 414. 1992. – Holotype: Perú. Piura, above Huancabamba, road to Piura, 3000 m, 10 Oct 1957, *P. C. Hutchison 1609* (US 00409556; isotype: F: V0076714F).

***Gynoxysinduta*** Cuatrec., Fieldiana, Bot. 27: 9. 1950. – Syntypes: Colombia. Valle, Cordillera Central, Hoya del río Bugalagrande, Barragán, Páramo de Bavaya, corrales, 3550–3400 m, 9 Apr 1946, *J. Cuatrecasas 20546* (COL: COL000005215COL000005216, F: V0076715FV0076716FV0076717F, P: P00711414, US 00122909, WIS: WIS00001047MAD).

***Gynoxysleiotheca*** S.F.Blake, J.Wash. Acad. Sci. 18: 35. 1928. – Holotype: Ecuador. Borma, Sep 1904, *Rivet 671* (P: P00711416; isotypes: US 00122915 (photo & fragments)).

***Gynoxyslittlei*** Cuatrec., Revista Acad. Colomb. Ci. Exact. 9: 242. 1954. – Holotype: Colombia. Huila, on foot of Cordillera Oriental, 20 km. SE of gigant, 103000 ft., 15 Sep 1944, *E. L. Little 8658* (F: V0076752F; isotypes: COL: COL000005220, US 00650427).

Note: This species is described as having “pale flowers” in the protologue; nonetheless, the label of the type specimen describes the flowers as “pale yellow”.

***Gynoxyslongifolia*** Wedd., Chlor. Andina 1(3): 79. 1855. – Syntypes: Perú. Cuzco, Andes de Cuzco, Oct 1839 – Feb 1940, *Gay s.n.* (F: V0076718F, P: P00711417P00711418P00711419, US 00122917 (fragments)).

***Gynoxyslopezii*** M.O.Dillon & Sagást., Brittonia 40(2): 223, f. 2. 1988 ≡ *Paragynoxyslopezii* (M.O. Dillon & Sagást.) Cuatrec., Phytologia 69(5): 314. 1990 ≡ *Paracalialopezii* (M.O. Dillon & Sagást.) A.Correa, Brittonia 55(2): 167. 2003. – Holotype: Perú. La Libertad, Patáz, Yaupa (Llaupa), entre Chagual-Retanas, carretera a Tayabamba, en borde carretera, pedregoso, 2300 m, 24 Jan 1974, *A. López & A. Sagástegui 8160* (HUT; isotypes: F: V0076719F, MO: MO–176388s.n.).

Note: The taxonomic assignment of this species was discussed by Cuatrecasas (1990) and Correa (2003). Its ratio of corolla lobe and tube length resembles the genus *Paracalia*, but we agree with Correa (2003) that this species belongs to *Gynoxys* because of its yellow flowers, shrubby (non-climbing) habit and central Andean distribution.

***Gynoxysmegacephala*** Rusby, Bull. New York Bot. Gard. 4: 398. 1907. – Syntypes: Bolivia. *M. Bang 1959* (F: V0076754F (fragments), GH: GH00008597, K: K000497526, MICH: MICH1107432, MO: MO–1183133, NY 178867178868, PH: PH00013514, US 00122920, WIS: WISv0256704WIS). Bolivia. Huaycani, 11000 ft., May 1866, *Pearce s.n.* (MO: s.n.).

= *Diplostephiumfoliosum* Rusby, Bull. New York Bot. Gard. 8(28): 128–129. 1912. – Syntypes: Bolivia. Cargadira, 8000 ft., 29 Jul 1902, *W. Roberts 1529* (BM: BM001024073, F: V0076745F, K: K000497534, NY 168221).

***Gynoxysmoritziana*** Sch.Bip. ex Wedd., Chlor. Andina 1: 79. 1855. – Syntypes: Venezuela. Merida, Sierra Nevada, 1844, *Moritz 1385* (GH: GH00008598GH00008599 (drawing & fragment), K: K000497525, P: P00711421P00711422P00711423, US 00122924 (fragments)).

***Gynoxyspendula*** Sch.Bip. ex Wedd., Chlor. Andina 1(3): 78. 1855. – Syntypes: Colombia. Nouvelle-Grenade, Mariquita, Boqueron, Tolima, Jan 1843, *J. J. Linden 954* (F: V0076760FV0076761F, GH: GH00008601, K: K000497523, NY 178870, P: P00711425P00711426, US 00122929 (fragments)).

= Gynoxyspendulavar.sinuata Cuatrec., Trab. Mus. Nac. Ci. Nat., Ser. Bot. 29: 38. 1935. – Syntypes: Colombia. Tolima, Andes, Cordillera Central, vert. merid. monte Tolima, loc. dict. Las Mesetas, 3600 m, 13 May 1932, *J. Cuatrecasas 2851* (MA: MA240997MA240997–2 (fragments)).

***Gynoxysregis*** H.Rob. & Cuatrec., Phytologia 56: 370(–371), f. 1984 ≡ *Paragynoxysregis* (H.Rob. & Cuatrec.) H.Rob. & Cuatrec., Novon 2(4): 415. 1992. – Holotype: Ecuador. Azuday, 30 km S of cumbé on the road to Saraguro at an elevation of 9800 ft., 26 Jan 1979, *R. M. King & F. Almeda 7804* (US 00122934; isotypes: K: K000497542, QCA: QCA17841).

***Gynoxyssoukupii*** Cuatrec., Bull. Soc. Bot. France 101: 245. 1954. – Holotype: Perú. Amazonas, Chachapoyas, cerro Puma Urco, Jun 1952, *Soukup 4072* (F: V0076774F; isotype: US 00122939).

***Gynoxyssubhirsuta*** Cuatrec., Notas Fl. Colombia 6: 35. 1944. – Holotype: Colombia. Santander, Cordillera Oriental, Páramo de Tamá, alrededores ed la Cueva, 3000–3200 m, 28 Oct 1941, *J. Cuatrecasas et al. 12714* (COL: COL000005229; isotypes: BC: BC634998, F: V0076778F, GH: GH00008611, U: U0001284, US 00122942).

#### ﻿*Gynoxys*, Praegynoxys group

***Gynoxysazuayensis*** Cuatrec., Brittonia 8(1): 39. 1954. – Holotype: Ecuador. Azuay, Eastern Cordillera, 4–6 km N of Sevilla de Oro, 9000–10000 ft., 16 Aug 1945, *Camp E–4724B* (F: V0076733F; isotypes: GH: GH00008572, K: K000497543, NY 178791, P: P00711396, US 00122890).

***Gynoxyscajamarcense*** (H.Rob. & Cuatrec.) B.Escobari & N.Kilian, **comb. nov.** ≡ *Aequatoriumcajamarcense* H.Rob. & Cuatrec., Novon 2(4): 411. 1992 ≡ *Nordenstamiacajamarcensis* (H.Rob. & Cuatrec.) B.Nord., Compositae Newslett. 44: 20. 2006. – Holotype: Perú. Cajamarca, Cutervo, Dist. San Andrés de Cutervo, Parque Nacional de Cutervo, caserío „Pajonal“ camino hacia Jaén, 2600 m, 10 Aug 1987, *Díaz & Osores 2585* (US 00409567; isotypes: F: V0043642F, MO: MO–2940604).

***Gynoxyscarpishensis*** Cuatrec., Brittonia 12: 185. 1960 ≡ *Aequatoriumcarpishense* (Cuatrec.) H.Rob. & Cuatrec., Novon 2(4): 412. 1992 ≡ *Nordenstamiacarpishensis* (Cuatrec.) B.Nord., Compositae Newslett. 44: 20. 2006. – Holotype: Perú. Carpish, between Huánuco and Tingo María, 2800 m, 10 Jul 1957, *H. Ellenberg 2211* (U: U.1610531; isotypes: GOET010400GOET010401).

***Gynoxyschingualensis*** H.Rob. & Cuatrec., Novon 2(4): 414. 1992. – Holotype: Ecuador. Sucumbíos, Paramo mirador SW of Playón de San Francisco, S del Río Chingual headwaters, 3400–3600 m, 15 May 1990, *P. King & Judziewicz 10131* (US 00409557; isotypes: F: V0076702F, K: K000497538, MO, S: S-R–2685).

***Gynoxyscongestiflora*** Sagást. & M.O.Dillon, Brittonia 37(1): 8, f. 3. 1985. – Holotype: Perú. Huánuco, ca. 46 Km NNE of Huánuco on road to Tingo María, Carpish Pass, E slope, 14 Jul 1981, *M. Dillon 2608* (F: V0043633F; isotypes: HUT, MO: MO–2940531, NY 178795, TEX00374263, US 00122897, USM: USM000112).

***Gynoxyscuatrecasasii*** B.Herrera, Bol. Soc. Perúana Bot. 8(1–2): 40, f. 30. 1980. – Holotype: Perú. Amazonas, Chachapoyas, Cerros Calla Calla, east side, 19 km. above Leimebamba on road to Balsas, 3100 m, 4 Jun 1964, *P. C. Hutchison & J. Kenneth Wright 5519* (USM: USM000114; isotypes: US 00122898, F: V0076740F, NY 804137).

***Gynoxysfabrisii*** Cabrera, Bol. Soc. Argent. Bot. 15(4): 332, f. 6. 1974. ≡ *Aequatoriumfabrisii* (Cabrera) C.Jeffrey, Kew Bulletin 47(1): 61. 1992 ≡ *Nordenstamiafabrisii* (Cabrera) B.Nord., Compositae Newslett. 44: 20. 2006. – Holotype: Argentina. Jujuy, Valle Grande, Serranía de Calilegua, senda Alto Calilegua, 2500 m, 18 Feb 1964, *H. A. Fabris et al. 5338* (LP: LP000275).

Note: Jeffrey (1992) and Nordenstam (2006) incorrectly cited a paratype as holotype and isotype.

***Gynoxysjaramilloi*** H.Rob. & Cuatrec., Novon 2(4): 415. 1992. – Holotype: Ecuador. Loja, Loma del Oro, 2800–3200 m, 4 Aug 1986, *Z. Jaramillo & Valencia 8799* (US 00409555; isotypes: MO: MO–1891634, QCA: QCA17836).

***Gynoxysjuninensis*** (H.Rob. & Cuatrec.) B.Escobari & N.Kilian, **comb. nov.** ≡ *Aequatoriumjuninensis* H.Rob. & Cuatrec., Novon 2(4): 412. 1992 ≡ *Nordenstamiajuninensis* (H.Rob. & Cuatrec.) B.Nord., Compositae Newslett. 44: 20. 2006. – Holotype: Perú. Junin, Carpata, above Huacapistana, 2700–3200 m, 7 Jun 1929, *Killip & Smith 24434* (US 00409566).

***Gynoxyskingii*** (H.Rob. & Cuatrec.) B.Escobari & N.Kilian, **comb. nov.** ≡ *Aequatoriumkingii* H.Rob. & Cuatrec., Novon 2(4): 412. 1992 ≡ *Nordenstamiakingii* (H.Rob. & Cuatrec.) B.Nord., Compositae Newslett. 44: 20. 2006. – Holotype: Bolivia. Cochabamba, 15 km from Colomi, on the road to Tunari, 10600 ft., 7 Feb 1978, *King & Bishop 7680* (US 00409565).

***Gynoxyslimonensis*** (B.Nord.) B.Escobari & N.Kilian, **comb. nov.** ≡ *Aequatoriumlimonensis* B.Nord., Compositae Newslett. 31: 14, f. 7. 1997 ≡ *Nordenstamialimonensis* (B.Nord.) B.Nord., Compositae Newslett. 44: 21. 2006. – Holotype: Ecuador. Morona-Santiago, 49 km from Limón on road to Gualaceo, 2300 m, 16 Jul 1996, *Stahl & Knudsen 2882* (S: S18–7653; isotype: QCA: QCA148693) .

***Gynoxyspascoensis*** (H.Beltrán & H.Rob.) B.Escobari & N.Kilian, **comb. nov.** ≡ *Aequatoriumpascoense* H.Beltrán & H.Rob., Compositae Newslett. 42: 5–7, f. 1. 2005 ≡ *Nordenstamiapascoensis* (H.Beltrán & H.Rob.) B.Nord., Compositae Newslett. 44: 22. 2006. – Holotype: Perú. Pasco, Oxapampa, trail to summit of Cordillera Yanachaga via Río San Daniel, 10°23’S, 75°27’W, 2600 m, 18 Jul 1984, *D. N. Smith & H. Botiger 7884* (USM; isotypes: AMAZ, MO: MO–037539, US 00810884).

***Gynoxysrepanda*** Wedd., Chlor. Andina 1(3): 77. 1855 ≡ *Aequatoriumrepandum* (Wedd.) C.Jeffrey, Kew Bull. 47(2): 292. 1992 ≡ *Nordenstamiarepanda* (Wedd.) Lundin, Compositae Newslett. 44: 16. 2006. – Syntypes: Bolivia. La Paz, Larecaja, Vallée de Tipuani, 1851, *M. Weddell s.n.* (F: V0076768F (fragments), P: P02273082, US 00122936 (fragments)).

Note: We consider the locality designation in the protologue “dans les taillis, sur le versant orientale du mont Illampù” to correspond to the (upper) Valle de Tipuani given on the label of the above specimen, because of its location east of Mt. Illampu. No specimen with the locality indication in the protologue could be found.

= *Schistocarphatriangularis* Rusby, Bull. New York Bot. Gard. 4: 392. 1907. – Syntypes: Bolivia. La Paz, Unduavi, Sep 1894, *M. Bang 2477* (F: V0076813F, GH: GH00549665, US 0012281900955547).

= *Gynoxysalternifolia* Sch.Bip. ex Rusby, Mem. Torrey Bot. Club 6(1): 67. 1896; Sch.Bip., Linnaea 34: 531. 1865, nom. nud. ≡ *Senecioalternifolius* (Sch.Bip. ex Rusby) Greenm., Ann. Missouri Bot. Gard. 10: 76. 1923. – Syntypes: Bolivia. La Paz, Vic. Mapiri, 8000 ft, Sep 1892, *Bang 1574* (A: A00008569, F: V0076725F, GH: GH00549664, K: K000634163, NDG: NDG62631, NY 114876114877, PH: PH00013520, PUL: PUL00000344, US 00122884). Bolivia. La Paz, Larecaja, Viciniis Sorata, inter Laripata et tani, in nemoribus, 3000–3200 m, Apr 1858–May 1859, *Mandon 131* (BR: BR0000005318605, GH: GH00012072, K: K000497519, MPU: MPU016063, P: P02273079P04099622P00711394P00711395).

***Gynoxysrimachiana*** Cuatrec., Phytologia 52(3): 164. 1982 ≡ *Aequatoriumrimachianum* (Cuatrec.) H.Rob. & Cuatrec., Novon 2(4): 413. 1992 ≡ *Nordenstamiarimachiana* (Cuatrec.) B.Nord., Compositae Newsletter 44: 22. 2006. – Holotype: Perú. Huanuco, Carretera de Tingo Maria - Huanuco, El Mirador, near Carpish, 2600–2700 m, 21 Mar 1980, *M. Rimachi 4908* (US 00324004; isotypes: F: V0043643F, US 00324003).

***Gynoxysstellatopilosa*** (Greenm. & Cuatrec.) B.Escobari & N.Kilian, **comb. nov.** ≡ *Seneciostellatopilosus* Greenm. & Cuatrec., Collect. Bot. (Barcelona) 3: 264. 1953 ≡ *Aequatoriumstellatopilosum* (Greenm. & Cuatrec.) C.Jeffrey, Kew Bull. 47(1): 62. 1992 ≡ *Nordenstamiastellatopilosa* (Greenm. & Cuatrec.) B.Nord., Compositae Newslett. 44: 22. 2006. – Holotype: Perú. Villcabamba, hacienda on río Chinchao, 6000 ft., 17 Jul 1923, *F. Macbride 4966* (F: V0043600F).

***Gynoxystovarii*** (H.Rob. & Cuatrec.) B.Escobari & N.Kilian, **comb. nov.** ≡ *Aequatoriumtovarii* H.Rob. & Cuatrec., Novon 2(4): 413. 1992 ≡ *Nordenstamiatovarii* (H.Rob. & Cuatrec.) B.Nord., Compositae Newslett. 44: 22. 2006. – Holotype: Perú. Huancavelica, Tayacaja, arriba de Marcavalle, entre Huachocolpa y Tintay, 3300 m, 21 Apr 1964, *O. Tovar 4781* (US 00409564).

***Gynoxystuestae*** (Cuatrec.) Cuatrec., Brittonia 8: 158. 1955 ≡ *Seneciotuestae* Cuatrec., Fieldiana, Bot. 27: 46. 1951 ≡ *Aequatoriumtuestae* (Cuatrec.) H.Rob. & Cuatrec., Novon 2: 413. 1992 ≡ *Nordenstamiatuestae* (Cuatrec.) B.Nord., Compositae Newslett. 44: 22. 2006. – Holotype: Perú. Huanuco, Pillao, 2700 m, 17 Feb 1946, *D. Tuesta Díaz & J. Woytkowski 34095* (F: V0043646F).

Note: This species is very likely conspecific with *G.repanda* and will be treated in a forthcoming work.

***Gynoxysvalenzuelae*** (H.Beltrán & J.Calvo) B.Escobari & N.Kilian, **comb. nov.** ≡ *Nordenstamiavalenzuelae* H.Beltrán & J.Calvo, Phytotaxa 474(3): 294, f. 1 & 2. 2020. – Holotype: Perú. Junín, Jauja, Monobamba, comunidad campesina Marancocha, zona de amortiguamiento del Bosque de Protección PuiPui, 11°18'39"S, 75°11'01"W, 3470 m, 25 Oct 2014, *L. Valenzuela et al. 28791* (USM: USM306000; isotypes: HOXA68690, MO: MO–2951169).

***Gynoxysvenezuelana*** (V.M.Badillo) B.Escobari & N.Kilian, **comb. nov.** ≡ *Aequatoriumvenezuelanum* V.M.Badillo, Ernstia, ser. 2, 10(1): 16, f. 9. 2000. – Holotype: Venezuela. Edo, Trujillo. Mun. Carache, Parque Nacional Dinira, arriba de Mesa, debajo del Pico Cendé, 9°53'N, 70°07'W, 3000 m, 1 Apr 1999, *Duno & Riina 783* (MY; isotype: VEN).

##### *Gynoxys* *s. l.*

***Gynoxysacostae*** Cuatrec., Feddes Repert. Spec. Nov. Regni Veg. 55: 129. 1953. – Holotype: Ecuador. Tunguragua, Alta de Pasa, 3500 m, 28 Oct 1944, *M. Acosta Solís 8738* (F: V0076722F).

***Gynoxysalbifluminis*** Cuatrec., Fieldiana, Bot. 27(2): 12. 1951. – Holotype: Perú. Lima, Río blanco, 15000 ft, 20 Mar 1923, *J. F. Macbride 3028* (F: V0076723F; isotype: US 00122883).

***Gynoxysalbivestita*** Cuatrec., Revista Acad. Colomb. Ci. Exact. 9: 242. 1954. – Holotype: Colombia. Boyacá, Nevada del Cocuy, Las Lagunillas, Pozo Azul, 4300 m, 12 Dic 1938, *J. Cuatrecasas 1434–A* (F: V0076724F; isotype: BC: BC624334).

***Gynoxysapollinaris*** Cuatrec., Fieldiana, Bot. 27(2): 16. 1951. – Holotype: Colombia. Caldas, Salamina, Corregimiento San Félix, Jul 1943, *T. Alberto 1884* (F: V0076726F; isotype: MEDEL: MEDEL000097).

***Gynoxysarnicae*** Cuatrec., Fieldiana, Bot. 27(1): 2–3. 1950. – Syntypes: Colombia. Departamento del Valle, Cordillera Occidental, Los Farallones, vertiente oriental, bajo el filo de la Cordillera en el cerro de La Torre: La Laguna, 3500–3550 m, 1 Aug 1946, *J. Cuatrecasas 21864* (COL: COL000005204COL000005205COL000005206, F: V0076728FV0076727F, K: K000497544, P: P00711392, US 00122885).

= Gynoxysarnicaevar.scandens Cuatrec., Fieldiana, Bot. 27: 3. 1950. – Syntypes: Colombia. Dep. del Valle, Cordillera Occidental, Los Farallones, extremo N. bajando a Las Cascadas, 3100 m, 2 Aug 1946, *J. Cuatrecasas 21923* (F: V0076729FV0076730F, P: P00711393, US 0012288600122887).

= Gynoxysarnicaef.subtomentosa Cuatrec., Fieldiana, Bot. 27: 3. 1950. – Syntypes: Colombia. Dep. del Valle, Cordillera Occidental, Los Farallones, lomas parameras sobre la mina El Diamante, 3000–3120 m, 31 Jul 1946, *J. Cuatrecasas 21834* (COL: COL000005203).

***Gynoxysasterotricha*** Sch.Bip., Linnaea 34: 529. 1865.

Lectotype (designated here): Bolivia. Larecaja, Viciniis Sorata, Lancha de Cochipata in scopulsis montis Illampia, 3300 m, 1 Apr 1859, *G. Mandon 84* (P02273125; isolectotypes: BR: BR0000005318506, F: V0076731FV0076732F, GH: GH00008570(!) GH00008571(!), MPU: MPU012549MPU012550MPU012570, NY 178790, P: P02273080(!) P02273126(!)).

Note: The gathering *Mandon 84* is a mixed collection of material representing *G.asterotricha* and *G.mandonii*. The above cited specimens in BR, F, GH, MPU, NY & P represent *G.asterotricha*. The specimen in K (K000497527) & P (P04099621(!)) holds material of both species on the same sheet.

***Gynoxysbaccharoides*** (Kunth) Cass. in Cuvier, Dict. Sci. Nat., ed. 2, 48(2): 455. 1827 ≡ *Seneciobaccharoides* Kunth, Nov. Gen. Sp. (folio ed.) 4: 146. 1818 [“1820”]. – Syntypes: Ecuador. Crescit locis frigidis Andium Quitensium, 3240 m, Jul, *F. W. H. A. Humboldt & A. Bonpland s.n.* (P: P00320174(!) P00320173(!)).

= *Gynoxyslindenii* Sch.Bip. ex Wedd., Chlor. Andina 1: 76. 1856. – Syntypes: Colombia. New Granada, Mariquita, Pic. de Tolima, 4280 m, *Linden 930* (syntypes; G: G00223897 F: V0076720F, NY 468695; US 00122916 (fragment)).

***Gynoxysbracteolata*** Cuatrec., Notas Fl. Colombia 6: 33, f. 26. 1944. – Holotype: Colombia. Caldas, Cordillera Central, vertiente occidental, faldas sudoese del Ruiz, El Aprisco, 3500–3600 m, 5 May 1940, *J. Cuatrecasas 9313* (COL; isotypes: BC: BC-Cuatrecasas–635016BC-Cuatrecasas–634964, F: V0076735FV0076734F, P: P00711406, US: US00122891, U: U 0001282).

***Gynoxysbuxifolia*** (Kunth) Cass. in Cuvier, Dict. Sci. Nat., ed. 2, 48(2): 455. 1827 ≡ *Seneciobuxifolius* Kunth., Nov. Gen. Sp. (folio ed.) 4: 147. 1818 [“1820”]. – Syntypes: Ecuador. Quito, Rucu Pichincha, Crescit cum praecedente: locis frigidis Andium Quitensium., *F. W. H. A. Humboldt & A. Bonpland s.n.* (F: s.n.V0077029F (fragments), HAL: HAL0113451, P: P00320176P00670367P00670368).

= Gynoxysbuxifoliavar.brevifolia Hieron., Bot. Jahrb. Syst. 19(1): 63. 1895. – Syntypes: Ecuador. Loja, Alsos de Zoghunes, Oña & Zaraguro, 3000–3300 m, 23 Oct 1888, *F. C. Lehmann 4899* (US 0012289201101244 (fragments), K: K000497541).

***Gynoxyscallacallana*** Cuatrec., Ciencia (Mexico) 23: 146. 1964. – Holotype: Perú. Amazonas, Chachapoyas, Middle eastern Calla-Calla slopes, ca. Kms. 411–416 of Leimebamba-Balsas road, 3100–3250 m, 11 Jul 1962, *J. J. Wurdack 1324* (US 00323999; isotypes: GH: GH00008574, LIMA, NY 178792, P, US 00811165).

***Gynoxyscalyculisolvens*** Hieron., Bot. Jahrb. Syst. 36: 504. 1905. – Syntypes: Perú. Cajamarca, entre Chota y Cutervo, Jun 1879, *C. von Jelski 611* (not traced), *C. von Jelski 780* (B, destroyed; photo: F: F0BN018153).

***Gynoxyscapituliparva*** Cuatrec., Fieldiana, Bot. 27(2): 6. 1951. – Holotype: Perú. Huanuco, Tambo de Vaca, 12000 ft., 10 Jun 1923, *J. F. Macbride 4434* (F: V0076736F; isotype: US 00122894).

***Gynoxyscaracensis*** Muschl., Bot. Jahrb. Syst. 50(2/3, Beibl. 111): 85–86. 1913. – Syntypes: Perú. Ancash, in declivibus Cordillerae blancae Supra Caraz, 3200–3700 m, 9 Jun 1903, *A. Weberbauer 3248* (B, destroyed). Perú. Ancash, Formatio aperta, 3600–3700 m, 18 Apr 1903, *A. Weberbauer 2909* (B, destroyed; photo: F: F0BN018154).

***Gynoxyscerrateana*** B.Herrera, Bol. Soc. Perúana Bot. 8(1–2): 37, f. 28. 1980. – Holotype: Perú. Amazonas, Chachapoyas, Cordillera Calla-Calla lado del Maranón, 3400–3600 m, *R. Ferreyra 15578* (USM).

***Gynoxyschagalensis*** Hieron., Bot. Jahrb. Syst. 28: 630. 1901. – Syntypes: Ecuador. Cuenca, chagal W Andens of Cutca, 2200–2800 m, Sep [no year], *F. C. Lehmann 7948* (B, destroyed; photo: F: F0BN018156; F: V0076703F, K: K000497539, US 00122895).

***Gynoxyschimborazensis*** Hieron., Bot. Jahrb. Syst. 29(1): 66. 1900. – Syntypes: Ecuador. Chimborazo, crescit in declivibus montis Chimborazo, 2600 m, Sep 1881, *A. Sodiro 60/9* (P: P00711408 (fragments), QPLS: QPLS211069).

***Gynoxyscolanensis*** M.O.Dillon & Sagást., Brittonia 40(2): 221. 1988. – Holotype: Perú. Bagua, Cordillera Colán, NE of La Peca , 78.26064N, 5.350383W, 2980–3100 m, 8 Sep 1978, *P. Barbour 3409* (F: V0043641F; isotypes: HUT, LSU: LSU00210549, MO: MO–2152935).

***Gynoxyscolumbiana*** (Klatt) Hieron., Bot. Jahrb. Syst. 28: 631. 1901 ≡ *Liabumcolumbianum* Klatt, Bot. Jahrb. Syst. 8(1): 47. 1886. – Syntypes: Columbia. Cauca, in silvis densis ad latera montis Páramo de Moras, 2800–3400 m, 16 Mar 1884, *F. C. Lehmann 3783* (GH: GH00008578 (fragment), K: K000497537, US 00122695).

***Gynoxyscompressissima*** Cuatrec., Fieldiana, Bot. 27(2): 4. 1951. – Holotype: Perú. Huanuco, Tambo de Vaca, ca. 12000 ft., 10–24 Jun 1923, *J. F. Macbride 4435* (F: V0076738F; isotype US 00122896).

***Gynoxyscorazonensis*** Hieron., Bot. Jahrb. Syst. 29: 65. 1900. – Syntype: Ecuador. Pichincha, Monte Corazón, *A. Sodiro 60/8* (P: P00711411 (fragments)).

***Gynoxyscostihirsuta*** Cuatrec., Ciencia (Mexico) 23: 146. 1964. – Holotype: Perú. Amazonas, Chachapoyas, upper slopes and summit of Cerro Yama-uma above Taulia, 12–15 km south-southeast (145°) of Molinopampa, 3200–3450 m, 11 Aug 1962, *J. J. Wurdack 1670* (US 00324000; isotypes: GH: GH00008580, K: K000497535, LIMA, LP: LP002068, NY 178796, P, US 00811164, USM: USM000113).

***Gynoxyscuicochensis*** Cuatrec., Fieldiana, Bot. 27: 16. 1951. – Holotype: Ecuador. Imbabura, Lake Cuicocha, 3500 m, 27 May 1939, *C. W. Pendland & R. H. Summer 722* (F: V0076705F).

***Gynoxyscusilluyocana*** Cuatrec., Fieldiana, Bot. 27(2): 8. 1951. – Syntypes: Perú. Cuzco, Paso de tres Cruces, Cerro de Cusilluyoc, 3500–3800 m, 3 May 1925, *F. W. Pennell 13900* (F: V0076741F, GH: GH00008582, PH: PH00013518, US 00122899).

***Gynoxyscutervensis*** Hieron., Bot. Jahrb. Syst. 36: 506. 1905. – Syntypes: Perú. Crescit prope Cutervo, May 1879, *C. von Jelski 632* (B, destroyed; photo: F: F0BN018157).

***Gynoxyscuzcoensis*** Cuatrec., Fieldiana, Bot. 27(2): 9. 1951. – Holotype: Perú. Cuzco, Tres Cruces, Pancartambo, 3600 m, 1 Oct 1941, *C. Vargas 2253* (NY 178797; isotypes: F: V0076742F (fragment), LP: LP002069LP002070).

***Gynoxyscygnata*** S.Díaz & A.Correa, Revista Acad. Colomb. Ci. Exact. 26(100): 343–344, f. 2. 2002. – Holotype: Colombia. Caldas, Sur del Nevado del Cisne, cerca a Laguna Verde, 04°50'07"N, 75°21'38"W, 4600–4800 m, 28 Jan 1986, *V.A.Funk 8082* (COL: COL000005207; isotype: US 01826640).

***Gynoxysdilloniana*** Sagást. & C.Téllez, Brittonia 39(4): 432, f. 1. 1987. – Holotype: Perú. Lambayeque. Ferreñafe, distrito Incahuasi, Laguna Tembladera-Cerro Negro, 3300 m, 12 Sep 1985, *A. Sagástegui et al. 12835* (HUT; isotypes: F: V0043636F, MO: s.n., NY).

***Gynoxysfallax*** Mattf., Repert. Spec. Nov. Regni Veg. 17: 183. 1921. – Syntypes: Perú. Piura, Huancabamba, westhänge der Cordillere östlich von Huancabamba, über der Hacienda Chantaco, 2500 m, 17 Apr 1912, *A. Weberbauer 6319b* (F: V0076706FV0076707F, GH: GH00008584).

***Gynoxysferreyrae*** B.Herrera, Bol. Soc. Perúana Bot. 8(1–2): 35. 1980. – Holotype: Perú. Cajamarca, Hualgayoc, Jalca, 16 Aug 1952, 3400 m, *R. Ferreyra 8559* (not traced; isotypes USM: USM000115; MO–714138, US 00122903).

Note: The protologue states the holotype specimen to be at USM (not traced online); the specimen in USM (USM000115) digitally available in JSTOR is labelled as isotype by Herrera. In case no other specimen exists in USM, USM000115 would actually be the holotype).

***Gynoxysflexopedes*** Cuatrec., Fieldiana, Bot. 27: 13. 1950. – Syntypes: Colombia. Cundinamarca, Paramo de Guasca, 3000–3500 m, 11 Oct 1939, H. *Garcia Barriga 08098* (COL: COL000005209COL000005208, F: V0076708FV0076709F, US 00122904).

***Gynoxysflorulenta*** Cuatrec., Fieldiana, Bot. 27(1): 4–5. 1950. – Syntypes: Colombia. Valle, Cordillera Central, Hoya del río Bugalagrande, Barragán, Páramo de Bavaya, Corrales, 3450–3520 m, 18–20 May 1946, *J. Cuatrecasas 20148* (COL000005210, COL000005211, F: V0076743FV0076744F, P: P00711412, US 00122905, WIS: WIS00001046MAD).

***Gynoxysfrontinoensis*** S.Díaz & A.Correa, Revista Acad. Colomb. Ci. Exact. 23(88): 333. 1999. – Holotype: Colombia. Antioquia, Municipio de Urrao, Páramo de Frontino, Llano Grande, 3460 m, 1 Jul 1984, *R. Lodo*ñ*o et al. 29* (COL: COL000005212; isotype: MEDEL: MEDEL000047).

***Gynoxysfuliginosa*** (Kunth) Cass. in Cuvier, Dict. Sci. Nat., ed. 2, 48(2): 455. 1827 ≡ *Seneciofuliginosus* Kunth, Nov. Gen. Sp. (folio ed.) 4: 146. 1818 [“1820”]. – Syntypes: Colombia. Pasto, Inter pagos Ypidales et Guachucal, 2916 m, Dic, *F. W. H. A. Humboldt & A. Bonpland s.n.* (F: V0076822F (fragments), P: P00320175P00670369).

= Gynoxysfuliginosavar.glabriuscula Domke, Biblioth. Bot. 116: 170. 1937. – Syntype: Ecuador. Cañar, Tipococha, 3200 m, 16 Aug 1933, *Diels 551* (B, destroyed).

***Gynoxyshuanucona*** (Cuatrec.) Cuatrec., Brittonia 8: 158. 1955 ≡ *Seneciohuanuconus* Cuatrec., Fieldiana, Bot. 27: 45. 1951 ≡ *Nordenstamiahuanucona* (Cuatrec.) B.Nord., Compositae Newslett. 44: 20. 2006. – Syntypes: Perú. Huanuco, 1927, *M. Sawada 45* (F: V0076921F, US 00123418).

***Gynoxyshallii*** Hieron., Bot. Jahrb. Syst. 19: 64. 1894. – Syntypes: Ecuador. Quito, crescit in regione suprema silvae Andinum occidentalium 2500–3400 m, Aug 1888, *F. C. Lehmann 4664* (K: K000634159K000634160); prope Zurucucho et Tambo de Quinua haud procul ab urbe Cuenca, 3000–3500 m, Sep 1888, *F. C. Lehmann 4605* (K: K000497532K000634158); In monte ignivomo Pichincha, 3400 m, *F. Hall s.n.* (B, destroyed; photo: F: F0BN018158).

***Gynoxyshenrici*** Mattf., Repert. Spec. Nov. Regni Veg. 17: 178. 1921. – Syntype: Perú. Amazonas, Östlich von Chachapoyas: zwischen dem steppe mit eizelnen Sträuchern, 3200–3400 m, 29 Jul 1904, *Weberbauer 4413* (B, destroyed).

***Gynoxyshirsuta*** Wedd., Chlor. Andina 1: 79. 1855. – Syntypes: Colombia. Bogotá, Nouvelle-Grenade, *F. W. H. A. Humboldt & A. Bonpland s.n.* (F: V0076746F, P: P00670371), *Goudot s.n*. (GH: GH00008586).

***Gynoxyshirsutissima*** Cuatrec., Notas Fl. Colombia 6: 34, f. 27–29. 1944. – Syntypes: Colombia. Cundinamarca, Cordillera oriental, extremo sudeste de la Sabana de Bogota en San Miguel, 2800–3000 m, 10 Sep 1941, *J. Cuatrecasas & R. Jaramillo 12022* (COL: COL000005213COL000005214, BC: BC–635006, F: V0076711FV0076712F, K: K000497530, LL: LL00374264, NY 178858, P: P00711413, U: U 0001283, US 00122902).

***Gynoxyshuasahuasis*** Cuatrec., Fieldiana, Bot. 27(2): 2. 1951. – Holotype: Perú. Huasahuasu, 2900 m, 29 Apr 1940, *F. Woytkowski 37* (F: V0076713F).

***Gynoxyshypoleucophylla*** Cuatrec., Ciencia (Mexico) 23: 148. 1964. – Holotype: Perú. Amazonas, Chachapoyas, Upper slopes and summit of Cerro Yamauma above Taulia, 12–15 km, south-southeast (145°) of Molinopampa, 3200–3450 m, 11 Aug 1962, *J. J. Wurdack 1671* (US 00324001; isotypes: GH: GH00008587, K: K000497529, LIMA, LP: LP002071, NY 178860, P, US 00811163).

***Gynoxysignaciana*** Cuatrec., Fieldiana, Bot. 27(2): 14. 1951. – Holotype: Ecuador. Pichincha, San Ignacio, 11200 ft., 14–19 Aug 1923, *H. E. Anthony & G. H. H. Tate 127* (US 00122908; isotype: F: V0076747F).

***Gynoxysinfralanata*** Cuatrec., Fieldiana, Bot. 27(2): 6. 1951. – Holotype: Perú. Cusco. Torontoy, Urubamba Valley, 3900 m, 1915, *E. Heller 2181* (US 00122910; isotype: F: V0076748F).

***Gynoxysjelskii*** Hieron., Bot. Jahrb. Syst. 36: 507. 1905. – Syntypes: Perú. Crescit prope Cutervo, May 1879, *C. von Jelski 678* (B, destroyed; photo: F: F0BN018159; F: V0076749F (fragments), US 00122912).

***Gynoxyslaurata*** Cuatrec., Fieldiana, Bot. 27: 5. 1950. – Syntypes: Colombia. Valle, Cordillera Central, cabeceras del río Tulu, quebrada de Las Vegas, 3400–3500 m, 23 Mar 1946, *J. Cuatrecasas 20399* (COL: COL000005217COL000005218COL000005219, F: V0076750FV0076751F, P: P00711415, US 00122913).

***Gynoxyslaurifolia*** (Kunth) Cass. in Cuvier, Dict. Sci. Nat., ed. 2, 48(2): 455. 1827 ≡ *Seneciolaurifolius* Kunth, Nov. Gen. Sp. (folio ed.) 4: 146. 1818 [“1820”]. – Syntypes: Ecuador. Loja, Crescit locis subcalidis, umbrosis inter Lucarque et Gonzanama Quitensium, 1908 m, Aug, *F. W. H. A. Humboldt & A. Bonpland s.n.* (B, destroyed; photo: F: F0BN018160).

***Gynoxyslehmannii*** Hieron., Bot. Jahrb. Syst. 28: 629. 1901. – Syntypes: Colombia. Cauca, crescit in fruticetis densis in Páramo de las Delicias in Andibus centralibus papayanensibus, 3200–3600 m, Jan-Feb, *F. C. Lehmann 8501* (B, destroyed; photo: F: F0BN018155; F: V0076721F, PH: PH00013515, S-R–2688, US 0012291401014476).

***Gynoxyslongistyla*** (Greenm. & Cuatrec.) Cuatrec., Chlor. Andina 1(3): 79. 1855 ≡ *Seneciolongistylus* Greenm. & Cuatrec., Collect. Bot. (Barcelona) 3: 292. 1953 ≡ *Nordenstamialongistyla* (Greenm. & Cuatrec.) B.Nord., Compositae Newslett. 44: 21. 2006. – Holotype: Perú. Moquegua, Saylapa near Carumas, 3600–3700 m, 3 Mar 1925, *Weberbauer 7331a* (F: V0076925F).

***Gynoxysmacfrancisci*** Cuatrec., Fieldiana, Bot. 27(2): 3. 1951. – Syntypes: Perú. Pachitea, Yanano, ca 6000 ft., 13–16 May 1923, *J. F. Macbride 3747* (F: V0076753F, US 00122918).

***Gynoxysmacrophylla*** Muschl., Bot. Jahrb. Syst. 50(2/3, Beibl. 111): 88–89. 1913. – Syntypes: Perú. Huanuco, Huamalies, Montes prope Monzon, 2000–2500 m, 8 Aug 1903, *Weberbauer 3534* (B, destroyed; photo: F: F0BN018161).

***Gynoxysmagnifolia*** (H.Beltrán & J.Campos) B.Escobari & N.Kilian, **comb. nov.** ≡ *Nordenstamiamagnifolia* H.Beltrán & J.Campos, Arnaldoa 16(1): 37. 2009. – Holotype: Perú. Amazonas, Luya. Camporredondo, Tullanga, Subiendo del campamento o Pascana hacia el Cerro Huicsocunga, 2700–3000 m, 7 Sep 1989, *C. Díaz & J.Campos 3830* (USM; isotypes: MO: MO–1962029MO–1962030, S: S19–3395S19–3398).

***Gynoxysmalcabalensis*** Cuatrec., Ciencia (Mexico) 23: 149. 1964. – Holotype: Perú. Amazonas, Chachapoyas, Summit of Cerro Malcabal (Cerro Tumbe) 3–6 km. southwest of Molinopampa, 2850–2900 m, 20 Jul 1962, *J. J. Wurdack 1413* (US 00324002; isotypes: GH: GH00008591, LIMA, LP: LP002072, NY 178862, P, USM: USM000117, US 00811161).

***Gynoxysmandonii*** Sch.Bip. ex Rusby, Mem. Torrey Bot. Club 6(1): 67. 1896; Sch.Bip., Bulletin de la Société Botanique de France 12: 80. 1865, nom. nud. – Lectotype (designated here): Bolivia. Cochabamba, Chapare, Espiritu Santo, 1891, *M. Bang 1196* (NY 178865; isolectotypes: BR: BR0000005318933, K: K000634162, NDG: NDG62632, PH: PH00013513). – Syntypes: Bolivia. Larecaja, Viciniis Sorata, Lancha de Cochipata in scopulsis montis Illampia, 3300 m, 1 Apr 1859, *G. Mandon 84* (BR: BR0000005317899, P: P00711420(!), S: S10–31297S10–31297, US 01117686).

= *Gynoxyshypomalaca* S.F.Blake, Bot. Gaz. 74: 427. 1922. – Holotype: Bolivia. La Paz, Sorata, higher limit of trees, 22 Apr 1920, *E. W. D. Holway & M. M. Holway 567* (US 00122907; isotypes: GH: GH00008588(!), NY 178861, US 01100708).

= *Gynoxyscochabambensis* Cabrera, Notas Mus. La Plata, Bot. 14: 194. 1949. – Holotype: Bolivia. Cochabamba, Chapare, Yanta-Aduana, 3200 m, 10 Jul 1929, *J. Steinbach 9813* (LP: LP000274; isotypes: E00414368, F: V0076737F, G: G00223898(!), GH: GH00008576GH00008577, K: K000634161K000659419, NY 178794, S: S-R–2686).

= *Gynoxyscruzensis* Cuatrec., Collect. Bot. (Barcelona) 3(3): 295. 1953. – Syntypes: Bolivia. Santa Cruz, Comarapa, Cerro San Mateo, 3400 m, 24 Oct 1928, *J. Steinbach 8515* (E00414367, F: V0076739F, GH: GH00008581, K: K000497536, PH: PH00013519, S: S-R–2687).

Note: The gathering *Mandon 84* is a mixed collection of material representing *G.asterotricha* & *G.mandonii*. The above cited specimens in BR, P, S, and US represent *G.mandonii*. The specimen in K (K000497527) depicts material of both ". The species are doubtfully distinct and will be treated in a forthcoming work.

***Gynoxysmarcapatana*** Cuatrec., Collect. Bot. (Barcelona) 3: 297. 1953. – Holotype: Perú. Cuzco, Quispicanchis, Marcapata, Compi-pampa, on the grade from Huaillai to Huallo-hualla, 4100 m, 11 Dic 1938, *C. Vargas 9717* (GH: GH00008596; isotype: F: V0076835F).

***Gynoxysmeridana*** Cuatrec., Bol. Soc. Venez. Ci. Nat. 15(81): 109. 1954. – Holotype: Venezuela. Merida, Laguna Negra, 9 Nov 1952, *L. Aristeguieta 970* (F: s.n.; isotypes: US 0012292100122922, VEN: VEN282322).

***Gynoxysmetcalfii*** Cuatrec., Fieldiana, Bot. 27(2): 2. 1951. – Holotype: Perú. Puno, Sandía. Near Limbani, 3200–3450 m, *R. D. Metfcalf 30529* (US 00122923).

***Gynoxysminiphylla*** Cuatrec., Fieldiana, Bot. 27(1): 11. 1950. – Holotype: Ecuador. Azuay, In vicinity of Toreador, between Molleturo and Quinoas, 3810–3930 m, 15 Jun 1943, *J. A. Steyermark 53175* (F: V0076701F; isotype: NY 178863).

***Gynoxysmonzonensis*** Mattf., Repert. Spec. Nov. Regni Veg. 17: 180. 1921. – Syntype: Perú. Huanuco, Huamalies, Berge südwestlich von Monzon, 3400–3500 m, 11 Jul 1903, *Weberbauer 3338* (B, destroyed).

***Gynoxysmultibracteifera*** H.Rob. & Cuatrec., Phytologia 56: 369, f. 1984. – Holotype: Ecuador. Azuay, Ridge between El Pan and Guachapala, 7500–9800 ft., 4 Sep 1945, *W. H. Camp E–5244* (US 00122925; isotype: NY 178864).

***Gynoxysmyrtoides*** Mattf., Repert. Spec. Nov. Regni Veg. 17: 182. 1921. – Syntype: Perú. Piura, Huancabamba, westhänge der Cordillere östlich von Huancabamba, über der Hacienda Chantaco, 5°10'W, 5°20'S, 2500 m, 17 Apr 1912, *Weberbauer 2. Ser., 6319a* (B, destroyed).

***Gynoxysneovelutina*** Cuatrec., Fieldiana, Bot. 27(2): 11. 1951. – Holotype: Bolivia, 3000 m, 1–4 Apr 1892, *O. Kuntze* (NY 178869; isotype: F: V0076755F (fragment)).

= *Gynoxystablaensis* Cabrera, Blumea 7: 197. 1952. – Syntypes: Bolivia. Cochabamba, Tablas, 3400 m, May 1911, *T. Herzog 2201* (B: B 10 0093559, L: L0001978L0001979, LP: LP000276, S: S-R–2690, Z: Z–000003473 (fragments)).

***Gynoxysnervosa*** Hieron., Bot. Jahrb. 21: 354. 1895. – Syntypes: Colombia. Boyacá, Crescit prope Muso civitatis Boyacá, Jul 1868, *A. Stuebel 161* (B, destroyed; photo: F: F0BN018162).

***Gynoxysnitida*** Muschl., Bot. Jahrb. Syst. 50(2/3, Beibl. 111): 86–87. 1913. – Syntypes: Perú. Ayacucho, Supra Quinuam prope Ayacucho, 3300–3500 m, 30 May 1910, *Weberbauer 5535* (F: V0076756F, G: G00223896 (fragments), GH: GH00008600, K: K000497524, US 00122927; photo: US 00122926).

***Gynoxysoleifolia*** Muschl., Bot. Jahrb. Syst. 50(2/3, Beibl. 111): 89–90. 1913. – Syntypes: Perú. Ancash, Pichiu, provinsia Huari, 4000–4100 m, 20. Apr 1903, *Weberbauer 2937* (photo F: V0076757F, S: S07–10464 (fragments)).

***Gynoxyspachyphylla*** Mattf., Repert. Spec. Nov. Regni Veg. 17: 184. 1921. – Syntype: Perú. Huancabamba, Cordillera östlich von Huacabamba, 5°20'S, 5°10'W, 3400–3500 m, 8 Apr 1912, *Weberbauer 2. Ser. 6082* (B, destroyed).

***Gynoxysparamuna*** Cuatrec., Fieldiana, Bot. 27: 7. 1950. – Syntypes: Colombia. Boyacá, Sierra Nevada del Cocuy, valle de Las Lagunillas, 4110 m, 11 Sep 1938, *J. Cuatrecasas & H. García Barriga 1434* (BC: BC–624335, COL: COL000005221, F: V0076758F, P: P00711424, US 00122928).

***Gynoxysparvifolia*** Cuatrec., Revista Acad. Colomb. Ci. Exact. 6: 59, f. 25. 1944. – Holotype: Colombia. Nariño, Páramo de la Laguna del Cumbal, 3475 m, 7 Feb 1942, *Miguel de Garganta 418* (COL: COL000005222; isotype: F: V0076759F).

***Gynoxysperbracteosa*** Cuatrec., Fieldiana, Bot. 27(1): 1. 1950. – Syntypes: Colombia. Cauca, Cordillera Central, Cabeceras del Río Páez, Páramo alrededor de la Laguna del Páez, 3450 m, 4 Dic 1944, *J. Cuatrecasas 19057* (COL: COL000005223COL000005224COL000005225, DUKE10000786, F: V0076762FV0076763F G: G00223895, GH: GH00008602, K: K000497522, MO: MO–714136, NY 178871, P: P00603125P00711427).

***Gynoxyspillahuatensis*** Cuatrec., Fieldiana, Bot. 27(2): 7. 1951. – Syntypes: Perú. Cuzco, „Pillahuata“, Cerro de Cusilluyoc, 3000–3300 m, 3 May 1925, *F. W. Pennell 14134* (F: V0076764F, GH: GH00008603, K: K000497521, NY 178872, PH: PH00013512, US 00122930).

***Gynoxyspoggeana*** Mattf., Repert. Spec. Nov. Regni Veg. 17: 179. 1921. – Syntypes: Perú. Junin, Valle del Río Masamerich, abajo del Tambo de Atac. 11°30'S, 3400–3500 m, 25 Apr 1913, *Weberbauer 2. Ser. 6645* (F: V0077103F, GH: GH00008605, MO: MO–714135 (fragments), MOL: MOL00006552, US 00122931, USM: USM000118).

***Gynoxyspsilophylla*** Klatt, Ann. K. K. Naturhist. Hofmus. 9: 367. 1894 ≡ *Gynoxysglabriuscula* Rusby, Mem. Torrey Bot. Club 6(1): 68. 1896, nom. illeg. – Syntypes: Bolivia. Cochabamba, 1 Jul 1891, *M. Bang 1116* (A: A00008585, BR: BR0000005318186BR0000005318513, E: E00413271, F: V0076765F, GH: GH00008606GH00008607, US 00122935, WIS: WISv0256703WIS).

= *Liabumbolivianum* Klatt, Ann. K. K. Naturhist. Hofmus. 9: 362. 1894 ≡ *Gynoxysboliviana* (Klatt) S.F.Blake, Contr. Gray Herb. 53: 28. 1918. – Holotype: Bolivia, *Cuming s.n.* (W: W18890106172; isotype: GH: GH00008573 (fragment with drawing)).

= *Gynoxyshoffmannii* Kuntze, Revis. Gen. Pl. 3(3): 156. 1898. – Syntype: Bolivia. Cochabamba, Weg zum Río Juntas, 3000 m, 13–21 Apr 1892, *O. Kuntze s.n.* (NY 178859).

***Gynoxyspulchella*** (Kunth) Cass. in Cuvier, Dict. Sci. Nat., ed. 2, 48(2): 455. 1827 ≡ *Seneciopulchellus* Kunth, Nov. Gen. Sp. (folio ed.) 4: 146–147. 1818 [“1820”]. – Syntype: Ecuador. Crescit locis frigidis Andium Quitensium.,3240 m, Jul, *F. W. H. A. Humboldt & A. Bonpland s.n.* (P: P00320177).

***Gynoxyspuracensis*** Cuatrec., Notas Fl. Colombia 6: 32. 1944. – Holotype: Colombia. Cauca, Cordillera Central, 2700–3100 m, 11 Jul 1939, *J. Cuatrecasas 5958* (COL; isotypes: BC: BC635070, F: V0076766F, P: P00711410; US 00122933).

***Gynoxysreinaldii*** Cuatrec., Fieldiana, Bot. 27(2): 15. 1951. – Holotype: Ecuador. Loja, Cajamuna, 2400 m, 7 May 1946, *R. Espinosa 312* (F: V0076767F).

***Gynoxysrimbachii*** Cuatrec., Fieldiana, Bot. 27: 10. 1950. – Syntypes: Ecuador. Eastern Cordillera, inner slope, 3200 m, Dec [no year], *A. Rimbach 79* (A: A00008608, F: V0076769F).

***Gynoxysrugulosa*** Muschl., Bot. Jahrb. Syst. 50(2/3, Beibl. 111): 87–88. 1913. – Lectotype (Herrera de Loja 1980: 39): Perú. Sandia, 3300 m, 11 Apr 1902, *Weberbauer 747* (F: F0BN018163 (photo)).

***Gynoxysrusbyi*** Cuatrec., Fieldiana, Bot. 27(2): 10. 1951. – Syntypes: Bolivia. La Paz, Vic. Pongo de Queme, 12500 ft., 2 Jul 1921, *H. H. Rusby 3* (F: V0076770F (fragments), MO: MO–1508476, NY 178874, US 00122937).

***Gynoxyssancti-antonii*** Cuatrec., Fieldiana, Bot. 27(1): 9. 1950. – Syntypes: Colombia. Comisaría del Putumayo, Páramo de San Antonio del Bordoncillo, entre el Encano y Sibundoy, 3250 m, 3 Jan 1941, *J. Cuatrecasas 11722* (BC: BC635012, COL: COL000005226, F: V0076771F, P: P00711429, US 00122938). Colombia. Narino, Yacuanquer, 2800–3000 m, 4 Jan 1943, *M de Garganta 504* (not traced).

= Gynoxyssancti-antoniivar.latifolia Cuatrec., Brittonia 12: 186. 1960. – Holotype: Ecuador. Chimborazo. Border to Canar (western escarpment), between Sta. Rosa and Joyagahi, 8000–9000 ft., *W. H. CampE–4049* (F: V0076772F; isotypes: GH: GH00008609, K: K000497518, MO: MO–714134, NY 178875, S: S-R–2689, VEN: VEN34425).

***Gynoxysseleriana*** Muschl., Bot. Jahrb. Syst. 50(2/3, Beibl. 111): 90–91. 1913. – Syntypes: Perú. Cuzco, Cazeo, in dumetis, 28 Jun 1910, *Seler 163* (B, destroyed). Perú. Cuzco, Urubamba, 3400 m, 10 Jun 1905, *Weberbauer 4926* (B, destroyed).

***Gynoxyssodiroi*** Hieron., Bot. Jahrb. Syst. 29: 64. 1900. – Syntypes: Ecuador. In decliv. m. Chimbor. vers. Guaranda, *L. Sodiro 60/3* (B, destroyed; photo: F: F0BN018164; QPLS: QPLS211119)

***Gynoxyssorataensis*** Cuatrec., Fieldiana, Bot. 27(2): 12. 1951. – Syntypes: Bolivia, La Paz, Sorata, 10000 ft, Feb 1886, *H. H. Rusby 1638* (F: V0076773Fs.n., MO: s.n., NY 178876).

***Gynoxysstuebelii*** Hieron., Bot. Jahrb. Syst. 21: 355. 1895. – Syntypes: Ecuador. Pichincha, Crescit prope Verdecuchu in monte Pichincha, 4000 m, Jul-Aug, *Stuebel 31* (B, destroyed; photo: F: F0BN018165). Ecuador. Pichincha, Monte Cayambe, 4300 m, *Stuebel 114* (not traced).

***Gynoxyssubamplectens*** Cuatrec., Fieldiana, Bot. 27(2): 1. 1951. – Syntypes: Perú. Cuzco, Paso de Tres Cruces, Cerro de Cusilluyoc, 3800–3900 m, 3 May 1925, *F. W. Pennell 13825* (F: V0076775F, GH: GH00008610, US 00122940).

***Gynoxyssubcinerea*** Cuatrec., Fieldiana, Bot. 27: 6. 1950. – Syntypes: Colombia. Santander, Cordillera Oriental, Hoya del río Chitagá en Vega Colombia, 2880 m, 28 Nov 1941, *J. Cuatrecasas 13473* (BC: BC634976, COL: COL000005227COL000005228, LP: LP002075, F: V0076776FV0076777F, P: P00711430, US 00122941).

***Gynoxysszyszylowiczii*** Hieron., Bot. Jahrb. Syst. 36(5): 505. 1905. – Syntypes: Perú. Caldas, Crescit prope Cutervo, May 1879, *Jelski 607* (B, destroyed); ibid., Apr 1879, *Jelski 754* (B, destroyed, photo: F: F0BN018166).

***Gynoxystabaconasensis*** H.Beltrán & S.Baldeón, Compositae Newslett. 47: 14, f. 1. 2009. – Holotype: Perú. Caldas, Province San Ignacio, District Tabaconas, Lagunas Arrebiatadas, Santuario Nacional Tabaconas-Namballe, 3150–3180 m, 9 Apr 2003, *S.Baldeón & L. Adrianzen 5160* (USM; isotypes: MO, S: S09–3275).

***Gynoxystetroici*** V.A.Funk & H.Rob., Revista Acad. Colomb. Ci. Exact. 17(65): 243–245, f. 1. 1989. – Holotype: Perú. Piura, Bosque de Huamba, 2950 m, 20 Sep 1987, *Valencia 1991* (US 00169692; isotype: USM).

***Gynoxystolimensis*** Cuatrec., Trab. Mus. Nac. Ci. Nat., Ser. Bot. 29: 37–38. 1935. – Syntypes: Colombia. Tolima, Cordillera Central, vert. merid. monte Tolima, El Salto, 3200 m, 15 May 1932, *J. Cuatrecasas 2850* (F: V0076779F (fragment), MA: MA240999).

***Gynoxystomentosissima*** Cuatrec., Ciencia (Mexico) 23: 149. 1964. – Holotype: Perú. Amazonas, Chachapoyas, middle eastern Calla-Calla, near Kms. 416–419 of Leimebamba-Balsas road, 3900–3100 m, 9 Jul 1962, *J. J. Wurdack 1254* (US00122943; isotypes: F: V0076780F, GH: GH00008612, K: K000497517, LP: LP002076, NY 178878, US 00811162, USM: USM000119).

***Gynoxystrianae*** Hieron., Bot. Jahrb. Syst. 21: 353. 1895. – Syntypes: Colombia. Nueva Granada, Tuquerres, 3000 m, Jun 1853, *Triana 1444* (B, destroyed; photo: F: F0BN018167; E: E00413269E00413270, NY 77375, P: P00711431P00711432, US 00122944). Colombia. Santisimo, haud procul a vico Cumbal, *Stuebel 435a* (not traced).

= Gynoxystrianaevar.nemocona Cuatrec., Fieldiana, Bot. 27(2): 17. 1951. – Holotype: Colombia. Cundinamarca, Nemocón, 2900–3000 m, 23 Oct 1917, *F. W. Pennell 2619* (NY 178879).

***Gynoxysvacana*** Cuatrec., Fieldiana, Bot. 27(2): 5. 1951. – Syntypes: Perú. Pasco, Tambo de Vaca, 13000 ft., 10–24 Jun 1923, J. F. Macbride 4391 (F: V0076782F, US 00122945).

***Gynoxysvalidifolia*** Cuatrec., Brittonia 8(1): 40. 1954. – Holotype: Ecuador. Azuay, N-NW of the Páramo del Castillo, 6–8 km N-NE of Sevilla de Oro, 10000–11200 ft., 31 Aug 1945, *W. H. Camp E–5156* (F: V0076783F; isotypes: GH: GH00008613, K: K000497516, NY 178880, US 00122946).

***Gynoxysvargasiana*** Cabrera, Revista Univ. (Cuzco) 33(87): 121–122, f. 20. 1944. – Holotype: Perú. Cuzco, Calvca, alrededores de Lares, 3200 m, 30 Aug 1943, *C. Vargas 3598* (LP: LP000277).

Note: Probably not a *Gynoxys.* Too many inner phyllaries for a *Gynoxys*.

***Gynoxysvenulosa*** Cuatrec., Fieldiana, Bot. 27(1): 8. 1950. – Syntypes: Colombia. Cauca, Cordillera Central, Cabeceras del Río López, Quebrada del Duende, 3400–3450 m, 3 Dic 1944, *J Cuatrecasas 18945* (COL: COL000005230COL000005231, DUKE: DUKE10000787, F: V0076784F, GH: GH00008614, K: K000497515, NY 178881, P: P00711433).

***Gynoxysviolacea*** Sch.Bip. ex Wedd., Chlor. Andina 1(3): 77. 1855. – Syntypes: Venezuela. Merida, Sierra nevada de Merida, 2920 m, 1 Sep 1846, *Funck & Schlim 1159* (F: V0076789F (fragments), GH: GH00008617 (fragments), K: K000497514, LD 1001683, MPU: MPU012551, P: P00711439P00711440P00711441P00711442, US 00122948 (fragments & photo)).

***Gynoxysvisoensis*** Cuatrec., Fieldiana, Bot. 27(2): 13. 1951. – Syntypes: Perú. Viso, 9000 ft., 5–14 May 1922, *Macbride & Featherstone 580* (F: V0076788F, US 00122949).

***Gynoxysweberbaueri*** Mattf., Repert. Spec. Nov. Regni Veg. 17: 181. 1921. – Syntype: Perú. Huancabamba, Cordillere östlich von Huancabamba, 3300–3500 m, 5°10'–5°20'S, 8 Apr 1912, *Weberbauer 2. Ser. 6075* (B destroyed).

***Gynoxyswoytkowskii*** (Cuatrec.) Cuatrec., Brittonia 8: 158. 1955 ≡ *Seneciowoytkowskii* Cuatrec., Fieldiana, Bot. 27: 49. 1951 ≡ *Nordenstamiawoytkowskii* (Cuatrec.) B.Nord., Compositae Newslett. 44: 22. 2006. – Holotype: Perú. Huánuco, vicinity of Tambo de Vacas, 3500 m, 4 Nov 1937, *F. Woytkowski 145* (F: V0076790F).

***Gynoxysyananoensis*** Cuatrec., Fieldiana, Bot. 27(2): 10. 1951. – Holotype: Perú. Huanuco, Yanano, 6000 ft., 20 Jun 1923, *J. F. Macbride 4931* (F: V0076791F)..

### ﻿Excluded names

*Gynoxysaquifolia* Cuatrec ≡ *Scrobicariaaquifolia* (Cuatrec.) B.Nord.

*Gynoxysauriculata* Turcz. = *Aetheolaenapatens* (Kunth) B.Nord.

*Gynoxysberlandieri* DC. = *Pseudogynoxyschenopodioides* (Kunth) Cabrera

*Gynoxyscordifolia* Cass. ≡ *Pseudogynoxyscordifolia* (Cass.) Cabrera

*Gynoxyscummingii* Benth. ≡ Pseudogynoxyschenopodioidesvar.cummingii (Benth.) B.L.Turner

*Gynoxysdiscolor* Rusby = *Pentacaliamarinii* (Cabrera) Cuatrec.

*Gynoxysfragrans* Hook. ≡ *Pseudogynoxysfragrans* (Hook.) H.Rob. & Cuatrec.

*Gynoxyshaenkei* DC. ≡ *Pseudogynoxyshaenkei* (DC.) Cabrera

*Gynoxysheterophylla* Turcz. ≡ *Aetheolaenaheterophylla* (Turcz.) B.Nord.

*Gynoxysilicifolia* (L.f.) Wedd. = *Scrobicariailicifolia* (L.f.) B.Nord.

*Gynoxysincana* Less. = *Jacmaiaincana* (Sw.) B.Nord.

*Gynoxyslaciniata* Less. = *Odontoclinelaciniata* (Sw.) B.Nord.

*Gynoxyslucida* Less. = *Dendrophorbiumlucidum* (Sw.) C.Jeffrey

*Gynoxysoerstedii* Benth. ≡ *Pseudogynoxysoerstedii* (Benth.) Cuatrec.

*Gynoxyspoeppigii* DC. ≡ *Pseudogynoxyspoeppigii* (DC.) H.Rob. & Cuatrec.

*Gynoxysprenanthifolia* Turcz. = *Aetheolaenapatens* (Kunth) B.Nord.

*Gynoxysscabra* Benth. ≡ *Pseudogynoxysscabra* (Benth.) Cuatrec.

*Gynoxyssinclairii* Benth. = *Pseudogynoxyssonchoides* (Kunth) Cuatrec.

### ﻿Names of doubtful status

For the following names in the Global Compositae Database, no publication is given:

*Aequatoriumcastillense* B.Nord.

*Aequatoriumstellatopilosum* Cuatrec.

*Gynoxysauriculata* Sch.Bip

*Gynoxyscumingii* Sch.Bip.

*Gynoxysglabrata* Less.

*Gynoxysincana* (Sw.) Griseb.

*Gynoxyslanceolata* Weddel

*Gynoxysnapoensis* H.Rob.

*Gynoxysperbracteata* Cuatrec.

*Gynoxysunduaviana* Cuatrec.

*Gynoxysverrucosa var. magna* Cuatrec.

## ﻿Additional information

### Author ORCIDs

Belen Escobari https://orcid.org/0000-0002-4588-3180

Tomas Borsch https://orcid.org/0000-0002-5724-2786

Norbert Kilian https://orcid.org/0000-0003-0890-4373

### Data availability

All of the data that support the findings of this study are available in the main text or Supplementary Information.

## Supplementary materials

Supplementary material 1 List of specimens investigated with full specimen data, lab codes and ENA accession numbers for molecular markers Data type xlsx https://binary.pensoft.net/file/919013This dataset is made available under the Open Database License (http://opendatacommons.org/licenses/odbl/1.0/). The Open Database License (ODbL) is a license agreement intended to allow users to freely share, modify, and use this Dataset while maintaining this same freedom for others, provided that the original source and author(s) are credited.Belen Escobari, Thomas Borsch, Norbert Kilian

Supplementary material 2 Majority consensus tree Data type docx Explanation note Majority consensus tree on a) Plastid genome (Escobari et al 2021), b) ITS, c) ETS, d) ETS-ITS concatenated under Bayesian inference topology. Posterior probability values. https://binary.pensoft.net/file/919014This dataset is made available under the Open Database License (http://opendatacommons.org/licenses/odbl/1.0/). The Open Database License (ODbL) is a license agreement intended to allow users to freely share, modify, and use this Dataset while maintaining this same freedom for others, provided that the original source and author(s) are credited.Belen Escobari, Thomas Borsch, Norbert Kilian

Supplementary material 3 Anchestral character analysis: Bayes Traits scores of the of morphological characters state shifts at the tree nodes of Fig. 5. Data type xlsx https://binary.pensoft.net/file/919015This dataset is made available under the Open Database License (http://opendatacommons.org/licenses/odbl/1.0/). The Open Database License (ODbL) is a license agreement intended to allow users to freely share, modify, and use this Dataset while maintaining this same freedom for others, provided that the original source and author(s) are credited.Belen Escobari, Thomas Borsch, Norbert Kilian
