# Supplementary material for: ﻿Generic concepts and species diversity within the Gynoxyoid clade (Senecioneae, Compositae)
Source: PhytoKeys. 2023 Oct 10;234:61–106. doi: 10.3897/phytokeys.234.107750 (PMC10582726; doi:10.3897/phytokeys.234.107750)
Supplement: Supplementary material 2 — Majority consensus tree [file phytokeys-234-061_article-107750__-s002.docx]

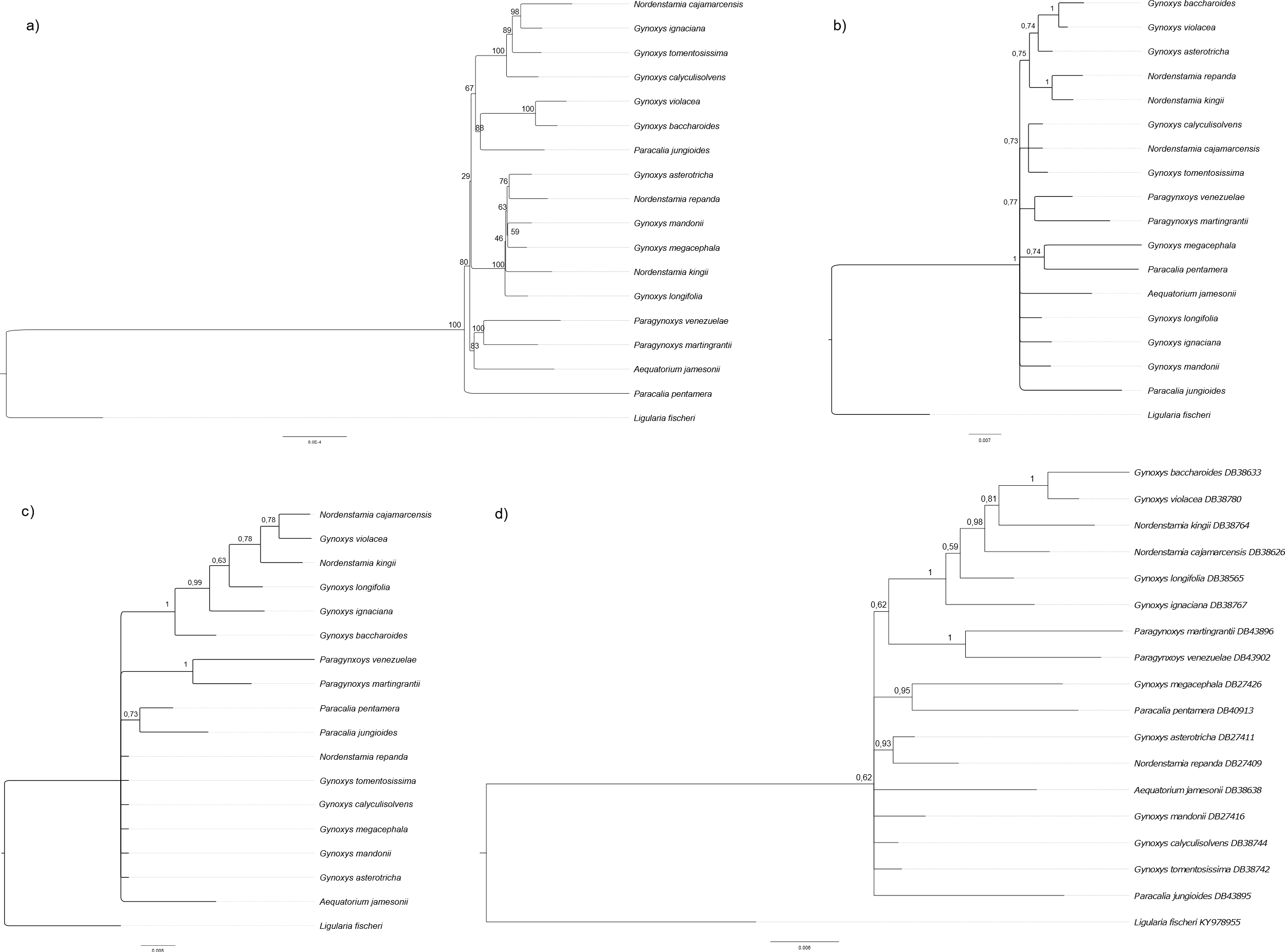
Appendix 2: Majority consensus tree on a) Plastid genome (Escobari et al 2021), b) ITS, c) ETS, d) ETS-ITS concatenated under Bayesian inference topology. Posterior probability values are given.
